# The Formose Reaction with SO_2_: A Computational Study

**DOI:** 10.3390/life16030513

**Published:** 2026-03-20

**Authors:** Emily M. Sisson, Jeremy Kua

**Affiliations:** Department of Chemistry & Biochemistry, University of San Diego, San Diego, CA 92110, USA

**Keywords:** origins of life, prebiotic chemistry, formose reaction, sulfur, proto-metabolism

## Abstract

This study examines the influence of SO_2_ and its hydrate H_2_SO_3_ on the free energies of the core autocatalytic cycle of the formose reaction. We find that SO_2_ and H_2_SO_3_ readily condense with aldehyde and alcohol functional groups to form bisulfite analogs of formose proto-metabolites under modeled conditions. The bisulfite functional group can provide intramolecular catalytic enhancement in specific isomers towards aldol additions and the retroaldol step that regenerates two equivalents of glycolaldehyde from tetrose. The bisulfite moiety reduces the favorability of the parasitic Cannizzaro side-reaction both thermodynamically and kinetically, thus potentially furnishing more throughput towards forming sugars. As a prebiotic analog to phosphate, we find that bisulfite slightly stabilizes ribose over its C_5_ aldose diastereomers thermodynamically, although the effect is modest and may be influenced by solution dynamics.

## 1. Introduction

The formose reaction is frequently invoked when discussing the chemical origins of life. It is a potentially autocatalytic source of a wide range of sugars including ribose and glucose, key building blocks in extant biochemistry. Autocatalytic chemical cycles are ubiquitous in living organisms [1], where they are embedded in compartments and under tight regulation, thus resilient to fluctuating environmental conditions. Such cycles are rare in “simple” chemical reactions which can be heavily influenced by an ever-changing environment. This is one reason why the core formose cycle has garnered interest as a potential model system to examine how modern metabolic cycles emerged from the seemingly chaotic chemistry of a proto-metabolism.

The formose reaction begins with formaldehyde (CH_2_O). As a C_1_ species, it is the “food” molecule or building block for constructing larger molecules, but requires an initiator to jumpstart oligomerization. The reaction is messy: CH_2_O oligomerizes through a series of aldol reactions generating sugars (CH_2_O)*_n_* of increasing size and diversity [2], but generates side-products such as carboxylic acids and polyols via parasitic Cannizzaro reactions. While the reaction mechanisms have been examined experimentally [3,4,5], there are many intricacies where varying experimental conditions result in different product distributions, which is why this complex system of reactions continues to be examined [6,7,8,9,10,11,12]. Several computational groups that have written code to model large-scale systems chemistry [13,14,15,16] chose to examine the formose reaction as a test case, highlighting its intriguing complexity.

**Figure 1:**
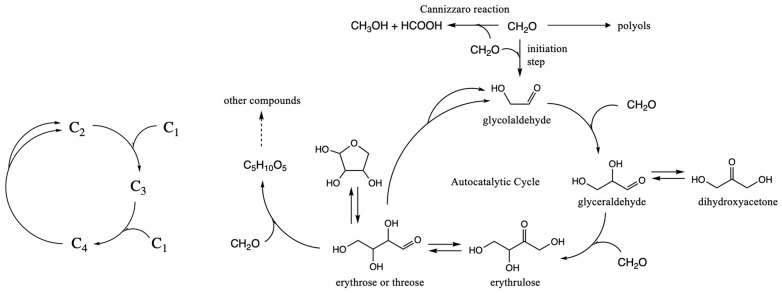
Core autocatalytic cycle of the formose reaction. Reproduced from our previous work [18] under the terms and conditions of the Creative Commons Attribution (CC BY) 4.0 license.

The core of the autocatalytic cycle nominally involves just three steps, represented by the simple abstract cycle on the left in Figure 1. The actual species involved are (CH_2_O)*_n_* molecules (*n* = 1–4), shown more explicitly on the right in Figure 1. If only CH_2_O is present initially, forming glycolaldehyde (C_2_H_4_O_2_) is very slow because C–C bond formation between two electrophilic carbons has a very high barrier [17]. However, if a small amount of this linchpin C_2_ species is formed or added as an initiator, the reaction proceeds rapidly via aldol reactions since an enol is now available as a nucleophile. The C_2_ glycolaldehyde reacts with CH_2_O to form C_3_ glyceraldehyde, which reacts further with CH_2_O to form C_4_ erythrulose. Autocatalysis is triggered if the C_4_ ketose isomerizes to an aldose which then generates two C_2_ molecules in a retro-aldol reaction; the increasing concentration of linchpin C_2_ rapidly eats up the C_1_ food as the cycle accelerates. The net reaction of this cycle is C_1_ + C_1_ → C_2_, not through the difficult direct dimerization, but rather facilitated by the C_2_, C_3_ and C_4_ species in a cycle of reactions. Larger sugars are formed through subsequent aldol reactions. Figure 1 shows one example (C_4_ + C_1_ → C_5_) but there are many other possibilities; we will explore the C_2_ + C_3_ → C_5_ reaction later in this article. While Figure 1 shows one example of the Cannizzaro reaction, note that all sugars present can potentially disproportionate into acids and polyols.

Extant biochemistry utilizes only a small subset of sugars while the formose reaction generates hundreds in an unregulated synthesis. Ribose, a crucial building block required in the RNA World hypothesis [19], is produced in very low yield under standard alkaline conditions of the formose reaction; it rarely accumulates even in the presence of catalysts. Many early experimental studies focused on tweaking the environmental conditions and adding catalysts/co-substrates to boost the yield of ribose; examples include borate [20], cyanamide [21], silicates [22], hydroxyapatite [23], and various metal catalysts [24]. Of particular interest to our present work is phosphate: Eschenmoser’s group found that the aldol addition of the glycolaldehyde phosphate and glyceraldehyde phosphate yielded ribose (with phosphates attached) in high yield over its diastereomers [25]. Subsequent work in the Lane group using acetylphosphate under formose conditions also favored ribose stabilization [26]. Phosphates play a critical role in extant life as evidenced by the ubiquity of kinases, enzymes that control biochemical reactions by phosphorylating and dephosphorylating a wide range of substrates.

However, one challenge is that under the reducing conditions of Hadean earth, soluble phosphate was rare [27,28]; although a recent suggestion is that dissolved Fe^2+^ may increase the availability of soluble phosphate [29]. On the other hand, the atmosphere of the early earth may have been rich with sulfur. A study of Hadean zircons suggests that redox conditions corresponded to the fayalite-magnetite-quartz buffer [30], thus implying that volcanic activity generated SO_2_, which hydrates to form H_2_SO_3_ when dissolved in aqueous solution. As a weak acid, H_2_SO_3_ generates bisulfite (and sulfite). Benner and co-workers prop [31] that prebiotic chemistry involving bisulfite adducts likely could have occurred given the production of SO_2_ from Hadean volcanoes. They also provide experimental evidence that formaldehyde-bisulfite adducts are stable, effectively sequester CH_2_O and reduce the parasitic Cannizzaro side reactions [32] in the formose system. Thus, we hypothesize that the presence of bisulfite adducts may provide some form of selectivity towards ribose and other biologically-relevant sugars.

This article explores the role of bisulfite by calculating the thermodynamics and kinetics of the early steps in the formose reaction in the presence of bisulfite adducts. We previously used a similar approach to study if thiols provide selectivity in the formose reaction [18]: Briefly, our results suggested that a key barrier for the C_2_ + C_1_ → C_3_ addition reaction was modestly lowered, which led to a pathway resulting in a marginally exergonic C_4_ → C_2_ + C_2_ retro-aldol reaction (but with Δ*G* very close to zero). In the absence of the thiol group, this retro-aldol reaction is slightly endergonic, which limits its autocatalytic potential. However the messiness and complexity of formose steps remained as isomeric reactions with the thiol in different positions led to a wide range of products, which does not tame the overall selectivity problem.

Could bisulfite analogs provide more favorable energetics to initiate autocatalysis? Our present work quantifies free energy changes (and associated activation barriers) for bisulfite adduct formation, aldol reactions of adducts in the formose cycle, and competing Cannizzaro side-reactions. We also investigate if sulfite could play the analogous role to phosphate in selecting ribose in the C_2_ + C_3_ → C_5_ aldol reaction. Computational studies such as ours are a static *model* system for a set of baseline conditions, and therefore our calculated free energies will not necessarily correspond to the dynamic conditions of an actual formose system under experimental conditions. Before getting into the results and discussion, we describe our computational protocol and its limitations.

## 2. Computational Methods

We use a similar computational protocol to our recent work on the formose cycle [33] and its thiol analogs [18]. This allows us to make direct comparisons and extend our free energy map. Here, we provide a brief description of that protocol for the convenience of our readers. Much of the text in this section (and the first two paragraphs of the next section) is reproduced from those two articles (also published in this journal) since we think the description is both clear and succinct. Our calculated free energies using this quantum chemistry protocol showed good agreement with available experimental results for CHO systems [17,34,35,36].

The computational details are as follows: The geometry of each molecule is optimized and its electronic energy calculated at the B3LYP (Becke3-Lee-Yang-Parr) [37,38,39,40] flavor of density functional theory with the 6-311G** basis set. To maximize the probability of finding global minima, multiple conformers are generated. The optimized structures are embedded in a continuum dielectric to calculate the aqueous solvation contribution to the free energy. While this does not provide a specific concentration, it assumes a dilute solution such that the electrostatic field generated by a neighboring solute molecule is effectively screened by a dielectric representing water using the SMD (Solvation Model based on Density) implicit solvent [41].

Zero-point energy corrections are included, and we apply the standard temperature-dependent enthalpy correction term (for 298.15 K) from statistical mechanics by assuming translational and rotational corrections are a constant times *kT*, and that low frequency vibrational modes generally cancel out when calculating enthalpy differences. However, entropic corrections in aqueous solution are problematic [42,43,44]. Changes in free energy terms for translation and rotation are poorly defined in solution due to restricted complex motion, particularly as the size of the molecule increases (thus increasing its conformational entropy). Free energy corrections come from two different sources: thermal corrections and implicit solvent. Neither of these parameters is easily separable, nor do they constitute all the required parts of the free energy. We follow the approach of Deubel & Lau [45], assigning the solvation entropy of each species as *half* its gas-phase entropy (calculated using standard statistical mechanics approximations similar to the enthalpy calculations described above), based on proposals by Wertz [46] and Abraham [47] that upon dissolving in water, molecules lose a constant fraction (~0.5) of their entropy.

To estimate activation energies, transition states were optimized by including several explicit water and/or catalytic molecules to facilitate proton transfer. All calculated transition states have one significant negative eigenvalue corresponding to the reaction coordinate. Each transition state was checked to ensure it had the correct eigenvectors for bond breaking/forming connecting the transition state to its reactants and products. Several conformers are tested, and for each reaction we only report the lowest calculated barriers with the optimum number of solvent molecules added to assist in proton transfer.

It is necessary to validate one’s computational protocol with available experimental values. Our protocol performs well comparing the equilibrium concentrations in a self-oligomerizing solution of 1 M glycolaldehyde at 298 K to subsequent NMR measurements [35]. Our relative Gibbs free energies in aqueous solution are typically within 0.5 kcal/mol/mol compared to experiment. That being said, our protocol has systematic errors of 2–3 kcal/mol/mol when calculating barriers involving carbonyl chemistry when compared to experimental results. Going to a higher level of theory does not reduce this error [48]; nor does using anionic species; our protocol shows better agreement with experimental results for the neutral forms [36]. Quantum chemistry is about error cancellation, and our protocol (with neutral molecules, implicit solvent, and a simplistic entropy correction) has worked well in the CHO systems we have studied even with a systematic error for activation barriers.

## 3. Results and Discussion

Throughout this paper, the thermodynamics of a chemical reaction are denoted by Δ*G*. For transition states, kinetic barriers are denoted by Δ*G*^‡^ and will refer to the forward barrier unless otherwise specified. A consistent set of reference compounds allows us to globally compare energies. In several Figures, *G*_rel_ values are found under each compound for local minima and in square brackets under an arrow for transition states. For a chemical reaction, Δ*G* = *G*_rel_(products) − *G*_rel_(reactants), and Δ*G*^‡^ compares *G*_rel_ of the transition state to either the reactants or products depending on whether the forward or reverse reaction is being discussed. The *G*_rel_ values for all compounds parsed into their separate enthalpic, entropic, and solvation free energy are provided in Supplementary Materials.

To connect this work to our prior thermodynamic map examining the formose cycle with H_2_S [18], we use a similar set of reference compounds: CO_2_, H_2_, H_2_O and H_2_SO_3_ are assigned a *relative* free energy, *G*_rel_ of 0.0 kcal/mol. Instead of H_2_S as our reference for thiols, we use the hydrate of SO_2_ as our reference for bisulfite analogs. Using the hydrate provided a cleaner comparison, and also better matched experimental thermodynamic values in previous work [36]. The *G*_rel_ values of all other species can be determined by calculating the change in free energy for *forming* the species, analogous to a free energy of formation. For example, the formation reaction of the bisulfite adduct of glycolaldehyde (C_2_H_4_SO_4_) is written as

2 CO_2_ + 4 H_2_ + H_2_SO_3_ → C_2_H_4_SO_4_ + 3 H_2_O

Since Δ*G* of this reaction is −12.8 kcal/mol, we assign *G*_rel_(C_2_H_4_SO_4_) = −12.8 kcal/mol. Because glycolaldehyde has *G*_rel_(C_2_H_4_O_2_) = −11.4 kcal/mol, we can quickly see that its bisulfite adduct is 1.4 kcal/mol more stable by comparing the two *G*_rel_ values. Note that the reaction shown above is to explain the thermodynamic reference states used to calculate *G*_rel_ and does not represent a prebiotic chemical reaction that actually takes place.

Our results begin with the addition of H_2_SO_3_ to form bisulfite analogs of C_1_ and C_2_ formose moieties. This is followed by examining the parasitic Cannizzaro reaction where bisulfite adducts reduce its favorability. We then examine how bisulfite analogs in the three steps of the smallest core formose cycle alter the relative kinetics and thermodynamics of the cycle. Finally, we explore the bisulfite analogs of the C_2_ + C_3_ → C_5_ aldol addition where ribose is marginally stabilized over its counterparts. A reminder that all our free energy values are for a model system under neutral conditions at 1 M in implicit solvent, absent catalysts, pH, or concentration changes. In the actual experimental system, these numbers will change. Therefore, while we present actual values of Δ*G* and Δ*G*^‡^, the reader should not interpret these as absolute values corresponding to an experimental system.

### 3.1. Initial Formation of Bisulfite Adducts and Rearrangements

While H_2_SO_3_ can form an adduct with any of the formose compounds, this section discusses the simplest cases: addition to formaldehyde (the C_1_ food supply) and glycolaldehyde (the linchpin C_2_ molecule for autocatalysis). Our calculated aqueous free energies establish the thermodynamics and kinetics of adding and rearranging bisulfite moieties; energetics of larger sugars are generally analogous and are discussed in their respective sections.

**Figure 2:**
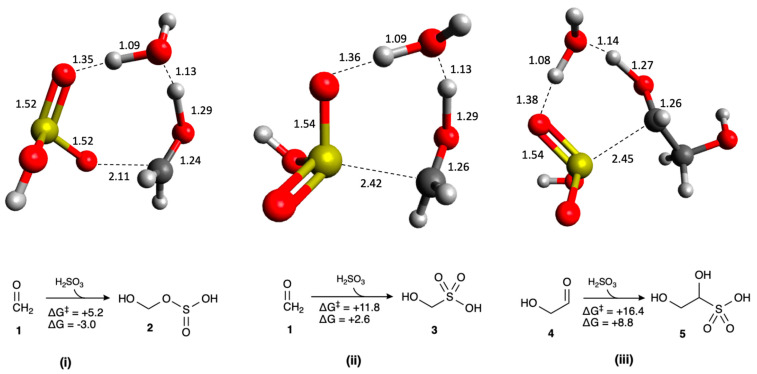
Reactions forming bisulfite adducts: **(i)** O-attack on CH_2_O, **(ii)** S-attack on CH_2_O, **(iii)** S-attack on glycolaldehyde (*G*_rel_ in kcal/mol, bond distances in Å).

We first consider the addition of H_2_SO_3_ to CH_2_O as shown in Figure 2. The carbonyl carbon of **1** (CH_2_O) is the electrophilic site. There are two possibilities: If the oxygen of H_2_SO_3_ acts as the nucleophile (“O-attack”), the reaction is slightly exergonic in our model (Δ*G* = −3.0 kcal/mol) with a low barrier (Δ*G*^‡^ = +5.2 kcal/mol) and forms the bisulfite adduct **2**. Alternatively, if the sulfur of H_2_SO_3_ acts as the nucleophile (“S-attack”), formation of the bisulfite adduct **3** is now thermodynamically unfavorable (Δ*G* = +2.6 kcal/mol) and the barrier has increased (Δ*G*^‡^ = +11.8 kcal/mol) although still kinetically feasible. The analogous S-attack on **4** (glycolaldehyde) to form the bisulfite adduct **5**, rightmost in Figure 2 is more unfavorable thermodynamically and kinetically. Transition states for all reactions are shown in Figure 2. We expect C_3_ and C_4_ S-attack adducts to be similarly unfavorable, and therefore the S-attack molecules will not be considered further in this article; we focus only on O-attack for adduct formation.

**Figure 3:**
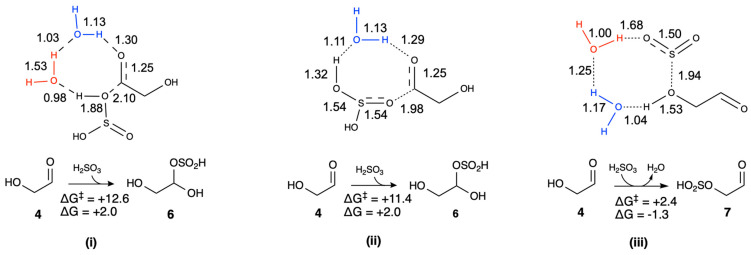
Three mechanisms for glycolaldehyde bisulfite adduct formation (*G*_rel_ in kcal/mol, bond distances in Å).

We now consider the addition of H_2_SO_3_ and SO_2_ to **4** in more detail. If bisulfite is the O-attack nucleophile attacking the carbonyl of glycolaldehyde, forming the adduct **6** is thermodynamically uphill (Δ*G* = +2.0 kcal/mol), although much less so than the S-attack. If the hydroxyl of H_2_SO_3_ performs the O-attack (reaction **(i)** in Figure 3), the barrier is relatively low (Δ*G*^‡^ = +12.6 kcal/mol), and the forming O…C bond is 2.10 Å in the transition state. If the sulfoxide (S=O) performs the O-attack (reaction **(ii)** in Figure 3), the barrier is marginally lower (Δ*G*^‡^ = +11.4 kcal/mol), and the forming O…C bond in the transition state is 1.98 Å. The optimal transition state in both cases is an 8-center transition state with relevant transition state distances schematically shown in Figure 3. More detailed transition state geometrical structures are shown in Supplementary Materials. As stated in Computational Methods, we try different numbers and geometries of catalytic water molecules when calculating transition states and only the lowest energy structures are shown.

If instead of reacting at the carbonyl end of glycolaldehyde, the hydroxyl of glycolaldehyde acts as the nucleophile and attacks the sulfur, this yields the bisulfite adduct **7** (reaction **(iii)** in Figure 3). The optimal transition state prefers H_2_SO_3_ to be dehydrated to an SO_2_ moiety in a 6-center transition state. The forming O…S bond is 1.94 Å. This reaction is marginally exergonic (Δ*G* = −1.3 kcal/mol) with a low barrier (Δ*G*^‡^ = +2.6 kcal/mol). We expect this pathway to be dominant since it is both thermodynamically and kinetically more favorable than the other two pathways.

**Figure 4:**
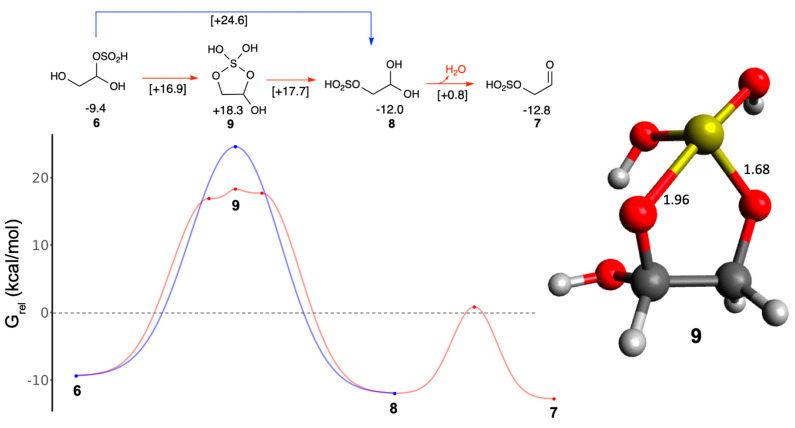
Rearrangement of glycolaldehyde bisulfite adduct. (*G*_rel_ in kcal/mol, bond distances in Å).

Next, we consider the rearrangement of adduct **6** to adduct **7**, which is thermodynamically favorable. It proceeds in two steps: the bisulfite group in **6** moves to the neighboring carbon forming adduct **8** before dehydrating to form **7** as shown in Figure 4. An energy curve is included to orient the reader to our use of *G*_rel_ values before we get to the complexity of the formose reactions. The *G*_rel_ of **6** and **8** are −9.4 and −12.0 kcal/mol respectively, therefore Δ*G* = −2.6 kcal/mol. Since *G*_rel_ of **7** is −12.8 kcal/mol, the dehydration of **8** to **7** is an additional 0.8 kcal/mol downhill. Thus, the rearrangement of **6** to **7** has overall Δ*G* = −3.4 kcal/mol. While the energy curve provides a visual aid and is easy to represent in a short linear pathway, such energy diagrams are difficult to represent for complex branching pathways we will examine later in this article. By providing *G*_rel_ values, the reader can get a quick sense of the global energy landscape.

For the bisulfite shift between **6** and **8**, we envisioned a transient intermediate with the ring structure **9**. Since *G*_rel_(**9**) is +18.3 kcal/mol, this intermediate is 18.3 − (−9.4) = 27.7 kcal/mol higher in energy than **6**. When we attempted to optimize the transition states on both sides of the intermediate, their energies were very similar (*G*_rel_ of +16.9 and +17.7 kcal/mol) and marginally *below* the intermediate. This suggests that our optimized structure **9** is not a stable intermediate and the transition state “hill” may be broad, as can be seen from the three red points on the energy diagram in Figure 4. The ring structure of **9** is asymmetric with S–O bond distances of 1.96 and 1.68 Å respectively. Since the two points on either side of **9** are similar in energy, this suggests that optimizing the transient ring intermediate can be used as a proxy to determine the bisulfite rearrangement barrier without having to optimize the more challenging transition states. We were able to optimize a symmetric transition state with a *G*_rel_ of +24.6 kcal/mol, but this is 7–8 kcal/mol higher (blue point on the hilltop in Figure 4) than the asymmetric structures. (Structures of all these transition states are in Supplementary Materials.) Regardless, rearrangement via a direct bisulfite shift has a much higher barrier and based on our results we hypothesize that adding and removing the SO_2_ moiety to be the predominant mechanism since it is kinetically facile with much lower barriers. Actual experimental environmental factor may change the preferred mechanism.

The adduct **7** with an aldehyde group is of similar thermodynamic stability to its hydrate **8** and the hydration barrier is ~13 kcal/mol. These values are very similar to our free energy calculations of glycolaldehyde and its hydrate without bisulfite, which closely matches experimental thermodynamic and kinetic values [35]. Whether **7** or **8** will be more favored will depend on how much water is present. In a dynamic fluctuating environment such as terrestrial hot spring wet-dry cycles proposed by Damer & Deamer [49], the relative abundances of different products will alternate with the cycles and the experimental free energy changes will differ from our calculated values.

### 3.2. Cannizzaro Reactions of C_1_ and C_2_ Bisulfite Adducts

**Figure 5:**
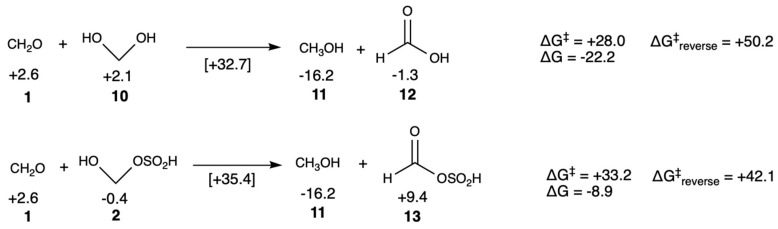
Cannizzaro reactions of formaldehyde and its bisulfite adduct (*G*_rel_ in kcal/mol).

The parasitic Cannizzaro disproportionation is always present in formose systems and detracts from the reactant pool by reducing the concentration of sugars participating in aldol reactions. While the uncatalyzed barrier is relatively high (Δ*G*^‡^ = +28.0 kcal/mol) for CH_2_O reacting with its hydrate **10** to form methanol **11** and formic acid **12** under neutral conditions, the reaction is highly exergonic (Δ*G* = −22.2 kcal/mol) as shown in Figure 5. Under alkaline conditions, the reaction would be kinetically much more feasible and Cannizzaro products build up [2]. (The Cannizzaro is thermodynamically strongly biased toward products under standard conditions; the reverse reaction is very endergonic and the reverse barrier would still be high even with a catalyst.)

In the previous section, we saw that bisulfite adds to formaldehyde to form adduct **2**. The Cannizzaro reaction of CH_2_O and **2** to form methanol and the corresponding bisulfite ester **13** is even more kinetically unfavorable under neutral or acidic conditions, and much less exergonic (Δ*G* = −8.9 kcal/mol). (See Supplementary Materials for the transition state structure.) While the barrier will be lowered in alkaline conditions the barrier with bisulfite is ~5 kcal/mol higher than without bisulfite. Thus, we expect that the presence of H_2_SO_3_ suppresses the Cannizzaro reaction. This was observed experimentally by Kawai et al. [32]; our results support their argument that adduct **2** provides a mechanism to sequester formaldehyde and suppress the likelihood of Cannizzaro reactions, but does not eliminate it entirely. However, sequestering formaldehyde will also slow down aldol additions involving CH_2_O.

**Figure 6:**
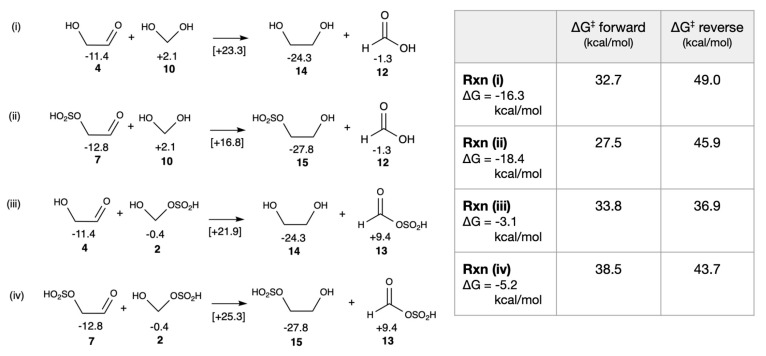
Cross-Cannizzaro reactions of glycolaldehyde and the bisulfite adduct of formaldehyde (*G*_rel_ in kcal/mol).

Although glycolaldehyde can self-disproportionate, its low concentration compared to formaldehyde in the early stages of the formose cycle makes this less likely. On the other hand, cross-Cannizzaro reactions between C_1_ and C_2_ aldehydes might occur. The results are shown in in Figure 6. For reaction **(i)** with no sulfur analog, Δ*G* = −16.3 kcal/mol and Δ*G*^‡^ = +32.7 kcal/mol under neutral conditions. If the bisulfite group is on glycolaldehyde and does not participate, as shown in reaction **(ii)**, Δ*G* is not too different although the barrier is lowered by ~5 kcal/mol. If the bisulfite group is on formaldehyde, the reaction is now much less exergonic, as shown in reactions **(iii)** and **(iv)**, with Δ*G* values of −3.1 and −5.2 kcal/mol respectively, as expected from our results in the previous section. This is because the favorable reduction of the C_2_ compound (forming **14** and **15**) is largely compensated by the unfavorable oxidation of **2** to **13**; the electron withdrawing bisulfite group disfavors its further oxidation in our computational model.

### 3.3. Glycolaldehyde-Bisulfite Reactions: Enolization, Aldol Addition and SO_2_ Elimination

**Figure 7:**
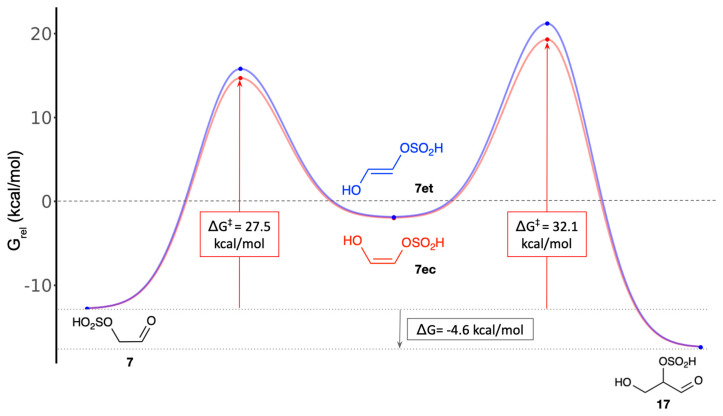
Enolization and C_2_ + C_1_ aldol addition of the bisulfite adduct.

As discussed in Section 3.1, the most stable glycolaldehyde bisulfite adduct is **7**, which is favorably generated by the addition of H_2_SO_3_ to glycolaldehyde. To participate in aldol reactions in the formose system, it must first enolize. The barrier to enolize under neutral conditions is relatively high (Δ*G*^‡^ = +27.5 kcal/mol), although under alkaline formose conditions, we hypothesize that this barrier would be substantially lowered. Forming the *cis*-enol is favored over the *trans*-enol both kinetically and thermodynamically, as shown in Figure 7. (**7ec** is 0.1 kcal/mol lower in energy than **7et**, benefiting from intramolecular hydrogen bonding.) An enediol is now susceptible to aldol addition with CH_2_O acting as the electrophile [50]. In the non-sulfur analog, the C_2_ + C_1_ aldol addition would form glyceraldehyde **16**; if the bisulfite group was retained throughout, the glyceraldehyde bisulfite adduct **17** is formed. As shown in Figure 7, the overall aldol addition is exergonic (Δ*G* = −4.6 kcal/mol) but the uncatalyzed barrier is high (Δ*G*^‡^ = +32.1 kcal/mol from the aldehydic reactants). In neutral aqueous solution in the absence of catalysts, the reaction is unlikely to occur.

**Figure 8:**
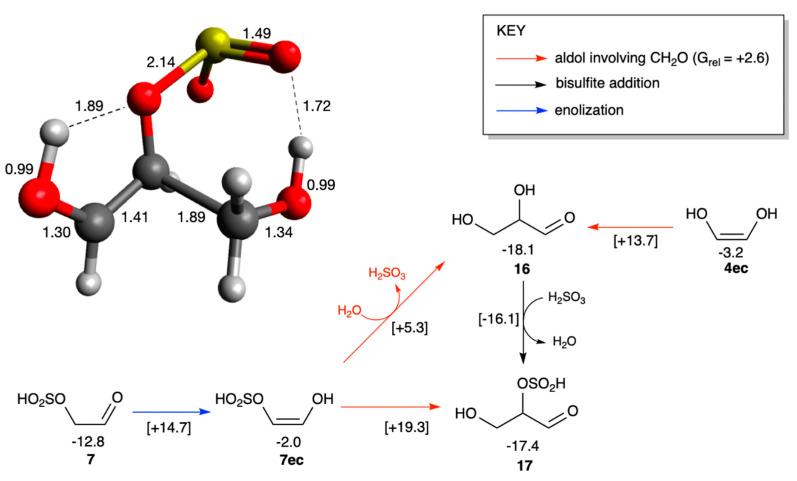
Competing aldol additions of the bisulfite enediol adduct (*G*_rel_ in kcal/mol, bond distances in Å).

The bisulfite moiety however provides an opportunity for intramolecular catalysis. When we tried to locate a transition state whereby the bisulfite acts as both proton donor and acceptor to facilitate the aldol addition, we always observed the elimination of SO_2_ concomitant with the aldol addition as shown in Figure 8 by the transformation of **7ec** + **1** to form **16**. The breaking O…S bond is 2.15 Å in the transition state, shown schematically in Figure 8 (see Supplementary Materials for transition state figures of both enolization and aldol addition steps). With intramolecular catalysis (and SO_2_ elimination), the barrier for aldol addition is reduced by 14 kcal/mol compared to **7ec** + **1** → **17** (the difference in *G*_rel_ of the two transition states with values of +5.3 and +19.3 kcal/mol). In comparison to the bisulfite-free analog, **4ec** + **1** → **16**, intramolecular catalysis lowers the barrier by ~8 kcal/mol. Because only a limited number of explicit solvent molecules are included in our transition states, these estimations are qualitative and in an actual system, SO_2_ elimination will depend on the pH and free energies of hydration.

As expected, addition of H_2_SO_3_ to glyceraldehyde (**16**) forming the adduct **17** is marginally endergonic (Δ*G* = +0.7 kcal/mol), not too different from what we found in the C_2_ case. The ~2 kcal/mol barrier is very low, thus adding and removing the bisulfite group is kinetically feasible and we expect additions and removals to be constantly taking place in a reaction mixture. Our findings suggest that the bisulfite moiety may play a potentially catalytic role in aldol reactions, at least in our model system.

### 3.4. Glyceraldehyde Bisulfite Reactions: Isomerizations and Aldol Additions

**Figure 9:**
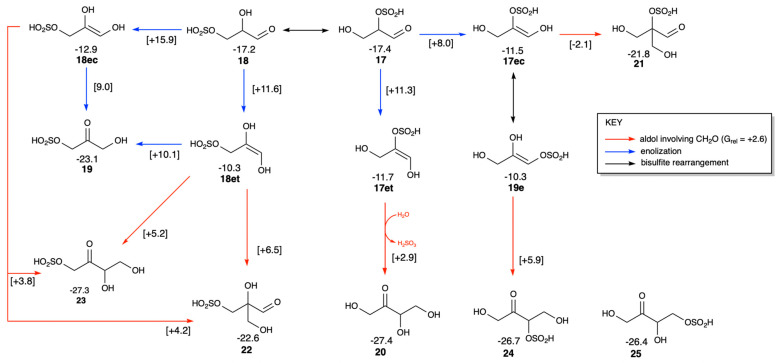
Isomerization and aldol addition of glyceraldehyde bisulfite adducts (*G*_rel_ in kcal/mol).

Since the bisulfite moiety can be easily added and removed, alongside adduct **17** we expect to also observe adduct **18** with bisulfite on the terminal rather than central carbon, as shown in Figure 9. They have similar stability with *G*_rel_ values of −17.4 and −17.2 kcal/mol respectively. In our model, the most likely mechanism for bisulfite rearrangement is removing SO_2_ in one position and adding it to the other. In previous sections, we saw that this route is kinetically much more feasible with very low barriers. Both aldehydes can enolize forming **17ec**, **17et**, **18ec** and **18et**. Not surprisingly, the thermodynamic sink of the trioses is dihydroxyacetone and its adduct **19** (enediol counterpart **19e**). In Figure 9, enolizations among C_3_ species have blue arrows, while bisulfite rearrangements have black arrows.

The C_3_ + C_1_ → C_4_ aldol additions in Figure 9 have red arrows. The most feasible is the intramolecularly catalyzed addition converting **17** to **20** (erythrulose), concomitant with SO_2_ removal. The overall reaction **17** + **1** + H_2_O → **20** + H_2_SO_3_ is exergonic with Δ*G* = −27.4 − (−17.4 + 2.6) = −12.6 kcal/mol; erythrulose is the thermodynamic sink of the tetroses. Similar to the C_2_ + C_1_ → C_3_ aldol addition discussed in the previous section, the presence of bisulfite lowers the aldol addition barrier substantially compared to its non-sulfur analog.) We were only able to find a transition state for the second step for the trans-enediol adduct **17et**, while its *cis* counterpart proceeded to form the branched aldose **21**.

While kinetically more favorable, the reaction **17** + **1** → **21** is much less exergonic with Δ*G* = −21.8 − (−17.4 + 2.6) = −7.3 kcal/mol, and is a dead-end reaction which cannot proceed to downstream products because **21** is non-enolizable. This non-productive reaction is therefore parasitic to the formose cycle, but will depend on the availability of enolizable species and the actual kinetics in aqueous solution. Its transition state (see Supplementary Materials) shows that bisulfite acts as an intramolecular proton shuttle, facilitating a lower barrier reaction. Similarly, **18** can form its corresponding branched aldose **22** (less sterically hindered than **21** and therefore marginally more stable by 0.8 kcal/mol). Since the bisulfite moiety is far from the active bond forming and breaking in the transition state and unable to participate in catalysis, its barrier is correspondingly higher. The reaction **18** + **1** → **22** is also parasitic to the formose cycle.

If the bisulfite adduct is not involved (and SO_2_ is not eliminated) in forming C_4_ ketose products, there are two more possibilities: **18** + **1** → **23** and **17** + **1** → **24**. The erythrulose adducts **23** and **24** have bisulfite at carbons 1 and 3 respectively. Both reactions are similarly exergonic (Δ*G* = −12.7 and −11.9 kcal/mol respectively) although they have higher barriers than the intramolecular catalyzed **17** + **1** + H_2_O → **20** + H_2_SO_3_ reaction discussed earlier. While there is no direct aldol addition route to ketose adduct **25** with bisulfite at carbon 4, removal and addition of SO_2_ should be facile. Since the *G*_rel_ values of **20**, **23**, **24**, and **25** are very similar, computationally this suggests a mixture of all these C_4_ ketoses may form at equilibrium; however kinetic factors and environmental conditions could skew the distribution. Of the three adducts, **23** (with bisulfite on carbon 1) is marginally more stable because of favorable intramolecular hydrogen bonding with the carbonyl.

### 3.5. Retro-Aldol Splitting of C_4_ Tetroses

**Figure 10:**
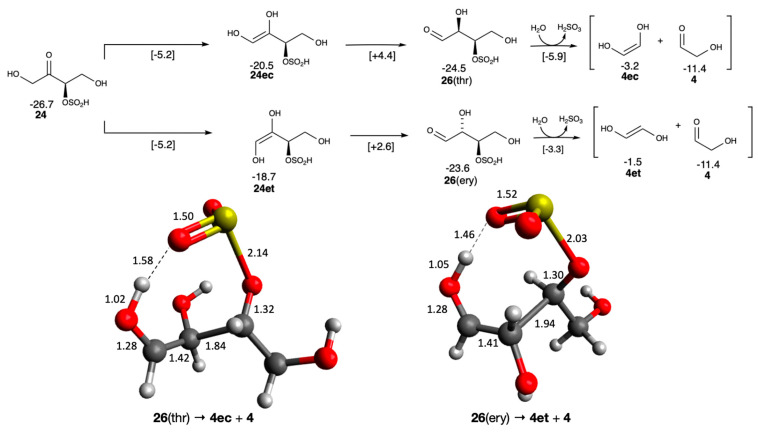
Enolization and retro-aldol reaction starting from the 3-bisulfite tetrose (*G*_rel_ in kcal/mol, bond distances in Å).

For autocatalysis to occur, two equivalents of glycolaldehyde must be regenerated in the key retro-aldol reaction C_4_ → 2 C_2_. The C_4_ ketose cannot do so without tautomerizing to an aldose, which may be unlikely at higher CH_2_O concentrations because of competing aldol additions [12]. Also, tautomerization can be slow, and kinetic competition depends on multiple environmental factors. Of the tetroses, only **24** can tautomerize without eliminating the bisulfite group as shown in Figure 10. There are two C_4_ aldose diastereomers, erythrose and threose, designated **26**(ery) and **26**(thr) respectively, with threose being marginally more stable by 0.9 kcal/mol. The initial barrier from **24** forming the *cis* and *trans* enols is similar in both cases (Δ*G*^‡^ = +21.5 kcal/mol), while the second step forming the aldose is marginally more favorable for erythrose compared to threose (the difference in *G*_rel_ of the two transition states with values of +2.6 and +4.4 kcal/mol). Only the biochemically relevant D-sugars are shown since their L-enantiomers should have similar physical properties.

Retro-aldol fragmentation of **26** via elimination of the bisulfite moiety leads to glycolaldehyde **4** and its enol (**4ec** or **4et**). Thermodynamically, this is marginally endergonic with Δ*G* of +2.5 and +3.3 kcal/mol for threose and erythrose, respectively. But the enols eventually convert to **4** (the rate of tautomerization will depend on actual experimental conditions); thus the overall reaction of **26** → **4** + **4** is exergonic with Δ*G* of −5.7 and −6.6 kcal/mol for threose and erythrose, respectively. In contrast, the non-sulfur analog retro-aldol C_4_ → 2 C_2_ is overall endergonic (post-tautomerization) with Δ*G* of +2.9 and +2.2 kcal/mol for threose and erythrose respectively [33]. In addition, the barrier with intramolecular bisulfite catalysis is modest (Δ*G*^‡^ of +18.6 and +20.3 kcal/mol for threose and erythrose respectively) compared to the high barrier ~32 kcal/mol in the non-sulfur analog based on our computational model. The transition states are shown in Figure 10; for threose the breaking C…C bond is 1.84 Å and the breaking S…O bond is 2.14 Å; meanwhile the H from bisulfite has hopped to the aldehyde oxygen. The analogous breaking C…C and S…O bonds are 1.94 Å and 2.03 Å, respectively, in erythrose.

The bisulfite retro-aldol reaction is also more favorable than the thiol analog, the latter being only marginally exergonic (Δ*G* = −0.2 kcal/mol) also with a high barrier of ~29 kcal/mol [18]. Thus, a key finding of this computational work is that bisulfite adducts lower barriers and make regeneration of the linchpin C_2_ more favorable under idealized conditions.

**Figure 11:**
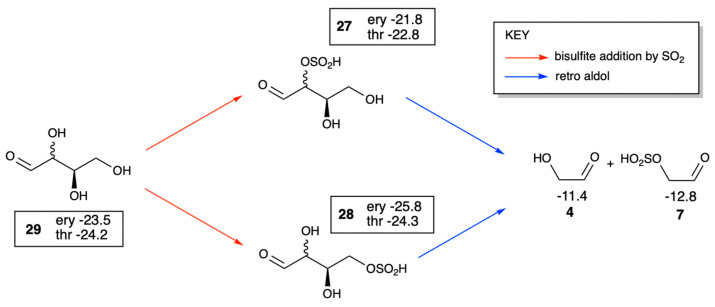
Enolization and retro-aldol reaction starting from the 3-bisulfite tetrose (*G*_rel_ in kcal/mol).

Since the bisulfite moiety can be easily added and removed, we also considered the isomers of adduct **26**. As shown in Figure 11, these isomers are **27** and **28** having bisulfite at carbon 2 and carbon 4 respectively. In our model, all three bisulfite adducts (**26**–**28**) would be in equilibrium with the non-sulfur analog **29**. The threose diastereomer is marginally more stable than erythrose in most cases. Of the three adducts, **28** is the most stable and sterically least hindered. The retro-aldol reactions of **27** and **28** do not require elimination of the bisulfite moiety, and they form the final products **4** and **7**. Thermodynamically, the reaction is marginally exergonic for **27** and marginally endergonic for **28** because of the relative stability of **28**. Since bisulfite does not act as an intramolecular catalyst in both cases, the barriers are expected to be high similar to the thiol or non-sulfur analogs.

The open-chain aldoses can also cyclize to form furanoses. This may sequester the aldose and lengthen its presence in the complex reaction mixture of a formose system. We found no significant effects for the bisulfite adducts, and our results shown in Supplementary Materials are essentially analogous to the non-sulfur analogs [33].

### 3.6. Thermodynamics of Aldopentose Formation

**Figure 12:**
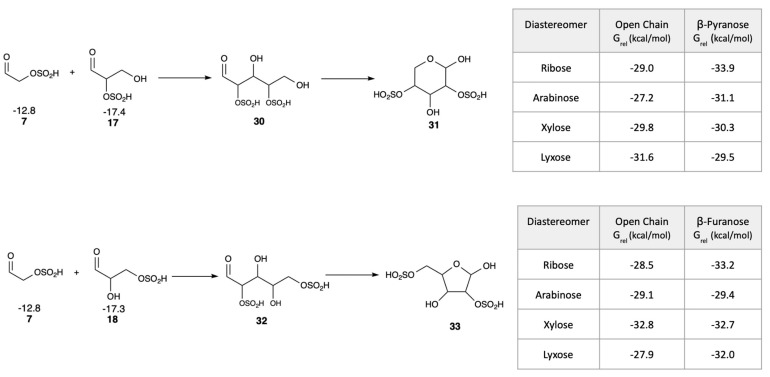
Thermodynamics of aldopentose formation and ring closure (*G*_rel_ in kcal/mol).

In the Introduction, we discussed experimental work on phosphate adducts, namely that aldol addition of glycolaldehyde phosphate and glyceraldehyde phosphate yielded ribose (with phosphates attached) in high yield over its diastereomers [25]. Could the bisulfite equivalent also favor ribose? Analogous to the experiments, we calculated the free energies of all four D-pentose diastereomers formed in the C_2_ + C_3_ → C_5_ aldol reaction with bisulfite in the 2 and 4 positions. (Mirror image L-enantiomers would have the same energies as their D-counterparts.) As shown in the top half of Figure 12, the reactants **7** and **17** have a combined *G*_rel_ of −12.8 − 17.4 = −30.2 kcal/mol. Forming the open chain 2,4-pentose adduct **30** is slightly uphill in three cases (and slightly downhill for lyxose with *G*_rel_ of −31.6 kcal/mol), but ring closure to the pyranose makes the overall reaction thermodynamically favorable for ribose, arabinose and xylose. Thus, our calculations suggest a tendency for cyclization but the actual efficiency will depend on experimental conditions. The 2,4-β-ribopyranose is the most stable diastereomer (*G*_rel_ of −33.9 kcal/mol), 2.8 kcal/mol more stable than arabinose. Based on our results, ribose appears slightly more stable than other diastereomers, but differences are within computational uncertainty especially comparing arabinose, xylose and lyxose. Comparing the pure sugars (no phosphate or bisulfite) in aqueous solution experimentally, arabinose would be favored over ribose by 0.8 kcal/mol [51]. Thus, the bisulfite moiety may play a similar role in favoring the 2,4-adduct over its diastereomers similar to phosphate.

For the 2,4-adduct, the bisulfite in the 4-position disfavors furanose formation, making pyranose dominant in our calculations. If instead the bisulfite was in the terminal position of glyceraldehyde (adduct **18**), the C_2_ + C_3_ aldol addition would yield the 2,5-pentose adduct **32** which can cyclize into the furanose **33** (while being prevented from forming the pyranose because of bisulfite in the 5-position). Forming the 2,5-adduct is slightly exergonic for xylose (*G*_rel_ of −32.8 kcal/mol) and marginally endergonic in the other three cases although ring closure to the furanose leads to 2,5-β-ribofuranose being thermodynamically the most stable diastereomer (*G*_rel_ of −33.2 kcal/mol). Xylofuranose, however, is less than 1 kcal/mol higher in energy, thus our results are less clear-cut in this case given the potential computational error.

Overall, our calculations suggest that bisulfite may provide similar thermodynamic stabilization as phosphate, and that ring formation in the aldopentoses helps drive thermodynamic favorability for the aldol addition, but experimental validation is needed. Also, since our calculated *G*_rel_ values are rather close to each other, this is not an overwhelmingly clear result.

## 4. Conclusions

Our computational study generating a free energy map for bisulfite adducts of the smallest autocatalytic cycle in the formose system was motivated by the proposal and previous experimental work by Benner and co-workers [31,32]. Within the limitations of our protocol, in our model system, we found that H_2_SO_3_ (the hydrate of SO_2_ in aqueous solution) easily attaches and detaches to both the aldehyde and hydroxyl groups on sugars. The barriers are low and the adducts are in equilibrium with the sugars and have similar *G*_rel_ values. Since adding the bisulfite moiety to the C_1_ food molecule (CH_2_O) is exergonic, this may reduce the effective concentration of free CH_2_O, thereby decreasing the likelihood of Cannizzaro reactions. This is because the favorable reduction half-reaction is largely compensated by the unfavorable oxidation half-reaction (due to the electron withdrawing bisulfite group on the species being oxidized). Our results also show that the presence of bisulfite increases the barrier by ~5 kcal/mol, kinetically disfavoring the Cannizzaro reaction although actual kinetic effects in complex prebiotic media may vary. This is in good agreement with experimental observations [32]; hence, the presence of SO_2_ may allow more throughput towards forming sugars and less leakage due to undesired side reactions.

With respect to the aldol additions and key retro-aldol reactions in the simplest formose cycle, our results suggest that intramolecular catalysis by bisulfite lowers the barrier in specific isomers; whether this translates to net autocatalytic flux depends on the relative populations of reactive species. In our optimized transition states, the S…O bond starts to break and the catalytic reaction involves elimination of SO_2_ from the adduct. A thermodynamic advantage is also observed for the retro-aldol reaction when bisulfite is removed from the tetrose, and the C_4_ → 2 C_2_ reaction is now exergonic unlike its non-sulfur analog. Hence, bisulfite adducts might allow for autocatalysis even at lower concentrations of tetrose, unlike the non-sulfur analog where a significant tetrose buildup is needed to overcome the endergonic retro-aldol step. The presence of SO_2_ might lead to autocatalysis being competitive in the formose system.

The bisulfite adducts of C_2_ and C_3_ could subsequently react and stabilize ribose over its diastereomeric counterparts. Computationally, bisulfite may modestly favor ribose over other pentoses, but in aqueous mixtures, this effect may be small relative to competing reactions. Like any other computational study, our results are limited by our protocol. We do not use a high-level density functional and basis set, we apply a simple entropy correction factor, and we use neutral compounds as proxies for what are likely to be anionic species under formose conditions. However, the results from our protocol compare favorably to experimental values in CHO systems, and we hope that extending it to sulfur-analogs will promote further experimental work in CHOS systems as potential proto-metabolites in origins-of-life studies.

## Data Availability

The data presented in this study are available in the article/Supplementary Materials. Further inquiries can be directed to the corresponding author.

## Supplementary Materials

The following supporting information can be downloaded at: https://www.mdpi.com/article/10.3390/life16030513/s1, Part 1: Energy breakdown of all molecules in this study, Part 2: Transition state structures, Part 3: Free energy comparison of open tetroses and ring structures, Part 4: XYZ coordinates for transition states.

## References

[1] Xavier J.C., Hordijk W., Kauffman S., Steel M., Martin W.F. (2020). Autocatalytic chemical networks at the origin of metabolism. Proc. Biol. Sci..

[2] Omran A., Menor-Salvan C., Springsteen G., Pasek M. (2020). The Messy Alkaline Formose Reaction and Its Link to Metabolism. Life.

[3] Breslow R. (1959). On the Mechanism of the Formose Reaction. Tetrahedron Lett..

[4] Appayee C., Breslow R. (2014). Deuterium studies reveal a new mechanism for the formose reaction involving hydride shifts. J. Am. Chem. Soc..

[5] Ricardo A., Frye F., Carrigan M.A., Tipton J.D., Powell D.H., Benner S.A. (2006). 2-Hydroxymethylboronate as a reagent to detect carbohydrates: Application to the analysis of the formose reaction. J. Org. Chem..

[6] Robinson W.E., Daines E., van Duppen P., de Jong T., Huck W.T.S. (2022). Environmental conditions drive self-organization of reaction pathways in a prebiotic reaction network. Nat. Chem..

[7] van Duppen P., Daines E., Robinson W.E., Huck W.T.S. (2023). Dynamic Environmental Conditions Affect the Composition of a Model Prebiotic Reaction Network. J. Am. Chem. Soc..

[8] Briš A., Baltussen M.G., Tripodi G.L., Huck W.T.S., Franceschi P., Roithova J. (2024). Direct Analysis of Complex Reaction Mixtures: Formose Reaction. Angew. Chem. Int. Ed..

[9] Paschek K., Kohler K., Pearce B.K.D., Lange K., Henning T.K., Trapp O., Pudritz R.E., Semenov D.A. (2022). Possible Ribose Synthesis in Carbonaceous Planetesimals. Life.

[10] Vinogradoff V., Leyva V., Mates-Torres E., Pepino R., Danger G., Rimola A., Cazals L., Serra C., Pascal P., Meinert C. (2024). Olivine-catalyzed glycolaldehyde and sugar synthesis under aqueous conditions: Application to prebiotic chemistry. Earth Planet. Sci. Lett..

[11] Haas M., Lamour S., Christ S.B., Trapp O. (2020). Mineral-mediated carbohydrate synthesis by mechanical forces in a primordial geochemical setting. Commun. Chem..

[12] Sutton S.M., Pulletikurti S., Lin H., Krishnamurthy R., Liotta C.L. (2025). Abiotic aldol reactions of formaldehyde with ketoses and aldoses—Implications for the prebiotic synthesis of sugars by the formose reaction. Chem.

[13] Rappoport D., Galvin C.J., Zubarev D.Y., Aspuru-Guzik A. (2014). Complex Chemical Reaction Networks from Heuristics-Aided Quantum Chemistry. J. Chem. Theory Comput..

[14] Simm G.N., Reiher M. (2017). Context-Driven Exploration of Complex Chemical Reaction Networks. J. Chem. Theory Comput..

[15] Roszak R., Wołos A., Benke M., Gleń Ł., Konka J., Jensen P., Burgchardt P., Zadlo-Dobrowolska A., Janiuk P., Szymkuc S. (2024). Emergence of metabolic-like cycles in blockchain-orchestrated reaction networks. Chem.

[16] Kan H.W., Li X.T., Zhang J.Z.H. (2025). Mechanistic insights into formose side reactions: Cannizzaro disproportionation and competing pathways. J. Chem. Phys..

[17] Kua J., Avila J.E., Lee C.G., Smith W.D. (2013). Mapping the Kinetic and Thermodynamic Landscape of Formaldehyde Oligomerization under Neutral Conditions. J. Phys. Chem. A.

[18] Kua J., Peña M.T., Cotter S.N., Leca J. (2025). Sulfur Analogs of the Core Formose Cycle: A Free Energy Map. Life.

[19] Neveu M., Kim H.J., Benner S.A. (2013). The “strong” RNA world hypothesis: Fifty years old. Astrobiology.

[20] Ricardo A., Carrigan M.A., Olcott A.N., Benner S.A. (2004). Borate minerals stabilize ribose. Science.

[21] Springsteen G., Joyce G.F. (2004). Selective derivatization and sequestration of ribose from a prebiotic mix. J. Am. Chem. Soc..

[22] Lambert J.B., Gurusamy-Thangavelu S.A., Ma K. (2010). The silicate-mediated formose reaction: Bottom-up synthesis of sugar silicates. Science.

[23] Usami K., Okamoto A. (2017). Hydroxyapatite: Catalyst for a one-pot pentose formation. Org. Biomol. Chem..

[24] Zubay G. (1998). Studies on the lead-catalyzed synthesis of aldopentoses. Orig. Life Evol. Biosph..

[25] Mueller D., Pitsch S., Kittaka A., Wagner E., Wintner C.E., Eschenmoser A. (1990). Chemistry of a-aminonitriles. Aldomerization of glycolaldehyde phosphate to rac-hexose 2,4,6-triphosphates and (in presence of formaldehyde) rac-pentose 2,4-diphosphates: Rac-allose 2,4,6-triphosphate and rac-ribose 2,4-diphosphate are the main reaction products. Helv. Chim. Acta.

[26] Camprubi E., Harrison S.A., Jordan S.F., Bonnel J., Pinna S., Lane N. (2022). Do Soluble Phosphates Direct the Formose Reaction towards Pentose Sugars?. Astrobiology.

[27] Gulick A. (1955). Phosphorus as a factor in the origin of life. Am. Sci..

[28] Keefe A.D., Miller S.L. (1995). Are polyphosphates or phosphate esters prebiotic reagents?. J. Mol. Evol..

[29] Brady M.P., Tostevin R., Tosca N.J. (2022). Marine phosphate availability and the chemical origins of life on Earth. Nat. Commun..

[30] Trail D., Watson E.B., Tailby N.D. (2011). The oxidation state of Hadean magmas and implications for early Earth’s atmosphere. Nature.

[31] Benner S.A., Kim H.J., Biondi E. (2019). Prebiotic Chemistry that Could Not Not Have Happened. Life.

[32] Kawai J., McLendon D.C., Kim H.J., Benner S.A. (2019). Hydroxymethanesulfonate from Volcanic Sulfur Dioxide: A “Mineral” Reservoir for Formaldehyde and Other Simple Carbohydrates in Prebiotic Chemistry. Astrobiology.

[33] Kua J., Tripoli L.P. (2024). Exploring the Core Formose Cycle: Catalysis and Competition. Life.

[34] Kua J., Hanley S.W., De Haan D.O. (2008). Thermodynamics and Kinetics of Glyoxal Dimer Formation: A Computational Study. J. Phys. Chem. A.

[35] Kua J., Galloway M.M., Millage K.D., Avila J.E., De Haan D.O. (2013). Glycolaldehyde Monomer and Oligomer Equilibria in Aqueous Solution: Comparing Computational Chemistry and NMR Data. J. Phys. Chem. A.

[36] Kua J., Hernandez A.L., Velasquez D.N. (2021). Thermodynamics of Potential CHO Metabolites in a Reducing Environment. Life.

[37] Vosko S.H., Wilk L., Nusair M. (1980). Accurate spin-dependent electron liquid correlation energies for local spin density calculations: A critical analysis. Can. J. Phys..

[38] Becke A.D. (1988). Density-functional exchange-energy approximation with correct asymptotic behavior. Phys. Rev. A.

[39] Lee C., Yang W., Parr R.G. (1988). Development of the Colle-Salvetti correlation-energy formula into a functional of the electron density. Phys. Rev. B.

[40] Becke A.D. (1993). Density-functional thermochemistry. III. The role of exact exchange. J. Chem. Phys..

[41] Marenich A.V., Cramer C.J., Truhlar D.G. (2009). Universal solvation model based on solute electron density and on a continuum model of the solvent defined by the bulk dielectric constant and atomic surface tensions. J. Phys. Chem. B.

[42] Warshel A., Florian J. (1998). Computer simulations of enzyme catalysis: Finding out what has been optimized by evolution. Proc. Natl. Acad. Sci. USA.

[43] Wiberg K.B., Bailey W.F. (2001). Chiral diamines 4: A computational study of the enantioselective deprotonation of Boc-pyrrolidine with an alkyllithium in the presence of a chiral diamine. J. Am. Chem. Soc..

[44] Nielsen R.J., Keith J.M., Stoltz B.M., Goddard W.A. (2004). A computational model relating structure and reactivity in enantioselective oxidations of secondary alcohols by (−)-sparteine-Pd(II) complexes. J. Am. Chem. Soc..

[45] Deubel D.V., Lau J.K. (2006). In silico evolution of substrate selectivity: Comparison of organometallic ruthenium complexes with the anticancer drug cisplatin. Chem. Comm..

[46] Wertz D.H. (1980). Relationship between the gas-phase entropies of molecules and their entropies of solvation in water and 1-octanol. J. Am. Chem. Soc..

[47] Abraham M.H. (1981). Relationship between solution entropies and gas phase entropies of nonelectrolytes. J. Am. Chem. Soc..

[48] Krizner H.E., De Haan D.O., Kua J. (2009). Thermodynamics and Kinetics of Methylglyoxal Dimer Formation: A Computational Study. J. Phys. Chem. A.

[49] Damer B., Deamer D. (2020). The Hot Spring Hypothesis for an Origin of Life. Astrobiology.

[50] Kim H.J., Ricardo A., Illangkoon H.I., Kim M.J., Carrigan M.A., Frye F., Benner S.A. (2011). Synthesis of Carbohydrates in Mineral-Guided Prebiotic Cycles. J. Am. Chem. Soc..

[51] Tewari Y.B., Goldberg R.N. (1985). An investigation of the equilibria between aqueous ribose, ribulose, and arabinose. Biophys. Chem..

