# Supplementary material for: The Formose Reaction with SO2: A Computational Study
Source: Life (Basel). 2026 Mar 20;16(3):513. doi: 10.3390/life16030513 (PMC13028158; doi:10.3390/life16030513)
Supplement: Supplementary file 1 [file life-16-00513-s001.zip › life-4200388-supplementary.pdf]

Supporting Information for:
The Formose Reaction with SO_2: A Computational Study
By Emily M. Sisson and Jeremy Kua*
University of San Diego, 5998 Alcala Park, San Diego, CA 92110, U.S.A.
*jkua@sandiego.edu

Part 1: Energy Breakdown for all molecules in this study

Table S1 contains the energy breakdown for calculated structures in this article. The computational methods section in the main text describes each of these categories: E_{elec} is the electronic energy in atomic units. All other values are in kcal/mol. E_{solv} is the solvation energy when the molecule is embedded in a dielectric. H_{corr} includes the zero-point-energy and standard enthalpy corrections to 298 K. The entropic correction (-$0.5TS_{corr}$) is half of the standard gas-phase entropy correction at 298 K. The last column (G_{298}) is the total free energy obtained by adding the other four columns (where the atomic units of E_{elec} are converted to kcal/mol by multiplying 627.5096. The transition states are labeled with a double-headed arrow between each structure. The negative frequency eigenvalue (in cm^{-1}) for each transition state is included in the table and the corresponding eigenvectors do correspond to the intrinsic reaction coordinate for the desired reaction.

Table S1. Energy breakdown of molecules and transition states.

	E_{elec} (a.u.)	E_{solv}	H_{corr}	$-0.5TS_{corr}$	G_{298}	G_{rel}
**Reference Molecules**						
H_2O	-76.44744	-9.75	15.75	-6.92	-47972.43	0.0
H_2	-1.17957	1.62	8.39	-4.64	-734.82	0.0
CO_2	-188.64114	2.10	9.60	-7.61	-118370.03	0.0
H_2SO_3	-625.10351	-13.91	23.73	-10.50	-392259.13	0.0
**C_1 molecules**						
**1** (formaldehyde)	-114.53634	-3.01	19.01	-7.99	-71864.64	2.6
**2**	-739.66660	-13.56	46.62	-11.96	-464126.79	-0.4
**3**	-739.64827	-19.49	46.69	-12.01	-464121.20	5.2
**10** (methanediol)	-190.99317	-15.48	37.44	-9.48	-119837.57	2.1
**11** (methanol)	-115.75739	-5.65	34.74	-8.46	-72618.25	-16.2
**12** (formic acid)	-189.81131	-12.46	23.62	-8.86	-119106.12	-1.3
**13**	-738.46587	-6.91	30.96	-11.76	-463382.13	9.4
**C_2 molecules**						
**4** (glycolaldehyde)	-229.11014	-8.70	41.61	-10.04	-143745.94	-11.4
**4ec**	-229.08972	-13.06	41.34	-10.00	-143737.72	-3.2
**4et**						
**5**	-854.21969	-19.86	69.06	-13.32	-535995.17	-1.5

**6**	-854.23270	-18.46	68.29	-13.66	-536003.03	-9.4
**7**	-777.77037	-12.22	49.13	-12.51	-488033.98	-12.8
**7ec**	-777.75366	-11.81	49.34	-12.88	-488023.24	-2.0
**7et**	-777.74754	-14.94	48.96	-13.11	-488023.14	-1.9
**8**	-854.24142	-16.09	68.48	-13.33	-536005.63	-12.0
**9**	-854.21846	-21.70	69.00	-13.25	-535996.24	-2.6
**14**	-230.31880	-13.23	57.33	-10.49	-144493.65	-24.3
**15**	-778.99135	-11.95	65.23	-12.56	-488783.83	-27.8
**C_3 molecules**						
**16**	-343.67607	-12.12	64.03	-11.72	-215619.85	-18.1
**17**	-892.33576	-14.33	71.62	-13.89	-559905.85	-17.4
**17ec**	-892.32582	-14.46	71.58	-14.01	-559899.91	-11.5
**17et**	-892.32371	-16.08	71.84	-14.18	-559900.11	-11.7
**18**	-892.33543	-14.08	71.53	-14.07	-559905.67	-17.2
**18ec**	-892.32816	-14.28	71.68	-14.27	-559901.36	-12.9
**18et**	-892.32049	-16.47	71.52	-14.14	-559898.76	-10.3
**19**	-343.67671	-15.04	63.53	-12.13	-215624.08	-22.3
**19e**	-892.32173	-15.86	71.75	-14.21	-559898.78	-10.3
**C_4 molecules**						
**20** (erythrulose)	-458.24446	-16.50	86.32	-13.41	-287496.38	-27.4
**21**	-1006.90501	-13.69	93.91	-15.14	-631777.48	-21.8
**22**	-1006.90447	-14.41	93.59	-15.25	-631778.29	-22.6
**23**	-1006.91081	-15.37	93.91	-15.35	-631783.01	-27.3
**24**	-1006.90429	-18.03	93.48	-15.74	-631782.40	-26.7
**24ec**	-1006.90228	-14.76	94.42	-15.05	-631776.23	-20.5
**24et**	-1006.89104	-19.24	94.05	-15.46	-631774.44	-18.7
**25**	-1006.91004	-15.20	93.96	-15.11	-631782.07	-26.4
**26**(thr)	-1006.90495	-16.41	93.87	-15.17	-631780.23	-24.5
**26**(ery)	-1006.90614	-14.90	93.98	-15.13	-631779.32	-23.6
**27**(thr)	-1006.89798	-18.68	93.78	-15.45	-631778.50	-22.8
**27**(ery)	-1006.90055	-15.78	93.60	-15.52	-631777.46	-21.8
**28**(thr)	-1006.90509	-16.18	93.91	-15.17	-631780.05	-24.3
**28**(ery)	-1006.90399	-17.55	93.45	-15.51	-631781.53	-25.8
**29**(thr)	-458.24338	-14.48	86.46	-13.10	-287493.24	-24.2
**29**(ery)	-458.24032	-14.95	86.07	-13.41	-287492.49	-23.5
**C_5 molecules**						
**30**(ribo)	-572.80174	-19.92	108.08	-14.91	-359365.34	-29.1
**30**(arab)	-572.80715	-17.93	108.52	-14.60	-359366.00	-29.7
**30**(xylo)	-572.80899	-16.74	108.51	-14.65	-359366.02	-29.8
**30**(lyxo)	-572.80476	-20.31	108.55	-14.60	-359366.84	-30.6
**31**(ribo)	-572.80620	-21.81	109.47	-14.16	-359367.89	-31.6
**31**(arab)	-572.80859	-20.54	109.38	-14.29	-359368.34	-32.1
**31**(xylo)	-572.80361	-23.87	109.61	-13.97	-359368.00	-31.7
**31**(lyxo)	-572.81259	-19.23	109.95	-13.75	-359368.43	-32.2
**32**(ribo)	-572.81237	-21.23	110.02	-13.86	-359370.33	-34.1
**32**(arab)	-572.80969	-22.52	109.97	-13.88	-359370.01	-33.8

**32**(xylo)	-572.80793	-23.49	109.93	-13.83	-359369.86	-33.6	
**32**(lyxo)	-572.80997	-21.62	109.93	-13.90	-359369.34	-33.1	
**33**(ribo)	-1670.12744	-21.74	123.05	-18.93	-1047938.63	-29.0	
**33**(arab)	-1670.12631	-22.55	122.43	-19.36	-1047939.78	-27.2	
**33**(xylo)	-1670.12622	-22.98	122.71	-18.96	-1047939.47	-29.8	
**33**(lyxo)	-1670.13195	-21.88	123.26	-18.76	-1047941.21	-31.6	
**34**(ribo)	-1670.13806	-22.39	124.40	-17.89	-1047943.55	-33.9	
**34**(arab)	-1670.13698	-20.52	124.61	-17.87	-1047940.77	-31.1	
**34**(xylo)	-1670.12684	-25.19	124.32	-18.46	-1047939.96	-30.3	
**34**(lyxo)	-1670.12929	-23.12	124.51	-18.38	-1047939.15	-29.5	
**35**(ribo)	-1670.12765	-20.70	122.83	-19.13	-1047938.13	-28.5	
**35**(arab)	-1670.12401	-24.17	123.10	-18.79	-1047938.71	-29.1	
**35**(xylo)	-1670.12426	-27.35	122.81	-18.90	-1047942.44	-32.8	
**35**(lyxo)	-1670.12943	-19.43	123.01	-18.93	-1047937.60	-27.9	
**36**(ribo)	-1670.12664	-27.40	123.73	-18.72	-1047942.90	-33.2	
**36**(arab)	-1670.12975	-22.52	124.17	-18.30	-1047939.10	-29.4	
**36**(xylo)	-1670.12596	-27.68	124.01	-18.65	-1047942.39	-32.7	
**36**(lyxo)	-1670.13622	-21.85	124.73	-18.02	-1047941.65	-32.0	
**Transition states**							Freq (cm^{-1})
**1** → **2**	-816.11000	-19.59	58.95	-13.46	-512090.96	7.8	-629.52
**1** → **3**	-816.08811	-26.11	58.57	-13.75	-512084.41	14.4	-807.76
**4** → **5**	-930.65582	-30.49	80.58	-15.71	-583961.08	5.0	-776.25
**4** → **6** mechanism **(i)**	-1007.14677	-25.47	99.39	-16.93	-631937.28	1.2	-275.18
**4** → **6** mechanism **(ii)**	-930.67307	-23.12	81.89	-15.14	-583962.66	3.4	-579.33
**4** → **7**	-854.21072	-21.78	62.53	-14.02	-535998.70	-5.1	-916.24
**6** → **9**	-930.65337	-22.55	81.63	-14.27	-583949.12	16.9	-558.22
**9** → **8**	-930.65773	-19.57	82.26	-14.38	-583948.35	17.7	-203.90
**8** → **7**	-1007.13900	-29.79	97.39	-15.88	-631937.67	0.8	-728.15
**6** → **8**	-1007.10662	-26.72	97.99	-16.07	-631913.88	24.6	-541.50
**1+10** → **11+12**	-381.96997	-15.13	70.81	-12.51	-239646.65	32.7	-1448.75
**1+2** → **11+13**	-930.61860	-21.05	77.81	-15.29	-583930.64	35.4	-1419.21
**4+10** → **14+12**	-496.53645	-22.11	94.11	-13.89	-311523.28	23.3	-1062.37
**7+10** → **15+12**	-1045.19828	-30.68	102.85	-16.73	-655816.52	16.8	-691.81
**4+2** → **14+13**	-1045.19251	-26.91	100.87	-17.03	-655811.41	21.9	-810.71
**7+2** → **15+13**	-1517.38596	-21.86	91.51	-17.69	-952122.29	25.3	-1070.59
**7** → **7ec**	-930.63933	-31.15	79.96	-15.03	-583951.33	14.7	-428.72
**7ec** → **16**	-892.28816	-20.06	70.51	-14.23	-559883.16	5.3	-218.25
**7ec** → **17**	-968.73238	-20.33	83.22	-15.63	-607841.61	19.3	-1042.79
**7** → **7et**	-930.63883	-27.68	77.64	-15.45	-583950.29	15.8	-1255.48
**7et** → **17**	-968.72996	-19.28	82.88	-15.91	-607839.66	21.2	-1158.12
**16** → **17**	-1045.25334	-28.41	102.47	-16.98	-655849.42	-16.1	-433.00
**4ec** → **16**	-420.08890	-15.29	77.16	-12.55	-263560.50	13.7	-823.53
**17** → **17ec**	-1045.20440	-34.56	101.97	-16.92	-655825.30	8.0	-375.64
**17** → **17et**	-1045.20302	-30.38	100.07	-16.80	-655822.04	11.3	-1277.42
**17** → **18**	-1045.19851	-27.82	100.83	-16.96	-655816.05	17.3	-589.05
**18** → **18ec**	-1045.19392	-32.09	100.79	-16.89	-655817.41	15.9	-1349.95

**18** → **18et**	-1045.20662	-29.56	101.85	-16.77	-655821.67	11.6	-405.95
**18ec** →**19**	-1045.21038	-30.26	102.15	-16.62	-655824.28	9.0	-584.55
**18et** → **19**	-1045.21188	-29.52	103.04	-16.27	-655823.24	10.1	-243.51
**17ec** → **19e** (ring proxy)	-892.27690	-16.59	71.06	-14.13	-559871.98	16.5	N/A
**17et** → **20**	-1083.30507	-28.64	105.48	-17.71	-679725.20	2.9	-642.06
**17ec** → **21**	-1006.85318	-24.28	92.06	-15.50	-631757.76	-2.1	-211.20
**18et** → **22**	-1159.77268	-28.99	121.61	-18.22	-727694.09	6.5	-939.77
**18ec** → **22**	-1159.76917	-31.56	120.58	-19.09	-727696.36	4.2	-1149.93
**18et** → **23**	-1083.31006	-26.89	108.20	-16.79	-679722.94	5.2	-331.79
**18ec** → **23**	-1159.78192	-26.62	122.39	-18.22	-727696.74	3.8	-432.05
**19e** → **24**	-1159.77147	-32.73	124.02	-18.17	-727694.61	5.9	-299.25
**24** → **24ec**	-1083.32462	-26.45	106.33	-16.60	-679733.32	-5.2	-989.30
**24ec** → **26**(thr)	-1236.25242	-29.84	141.39	-19.89	-775668.60	4.4	-599.98
**26**(thr) → **4ec**+**4**	-1006.85898	-24.09	91.89	-15.69	-631761.57	-5.9	-271.01
**24** → **24et**	-1083.31978	-29.47	106.34	-16.59	-679733.28	-5.2	-1108.14
**24et** → **26**(ery)	-1236.24886	-34.08	141.04	-19.33	-775670.40	2.6	-456.92
**26**(ery) → **4et**+**4**	-1006.84221	-31.43	91.46	-15.84	-631758.96	-3.3	-407.93
**27**(ery) → **4**+**7et**	-1159.76086	-38.23	122.26	-19.01	-727696.05	4.5	-493.95
**27**(thr) → **4**+**7ec**	-1159.76588	-31.79	121.25	-19.06	-727693.83	6.7	-861.34
**28**(ery) → **4et**+**7**	-1159.77890	-38.20	123.50	-18.02	-727705.11	-4.6	-399.08
**28**(thr) → **4ec**+**7**	-1159.77288	-32.03	123.84	-18.61	-727695.42	5.1	-229.20
**30**(rib) → **31**(rib)	-1823.02039	-34.09	152.53	-21.01	-1143865.37	-10.9	-915.54
**32**(rib) → **33**(rib)	-725.70839	-29.86	137.64	-16.48	-455297.68	-16.6	-991.28

Part 2: Transition State Structures

Figures S1 through S6 contained more detailed transition state structures described in the main paper but not shown. These structures were generated with Avogadro. All bond distances are in Å and all energies are in kcal/mol.

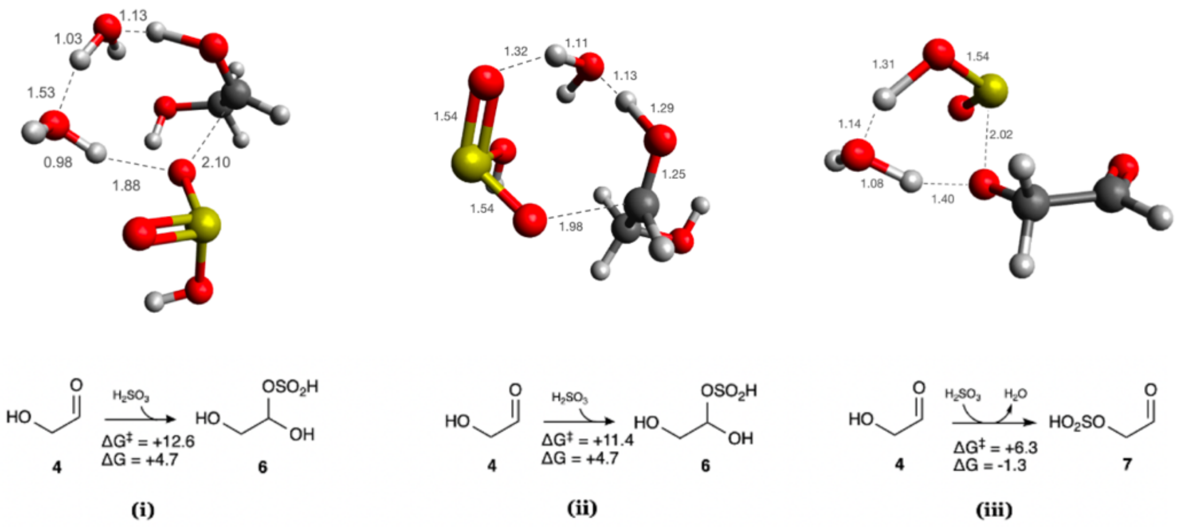

Figure S1. Transition states of H_2SO_3 addition to glycolaldehyde (various O-attacks).

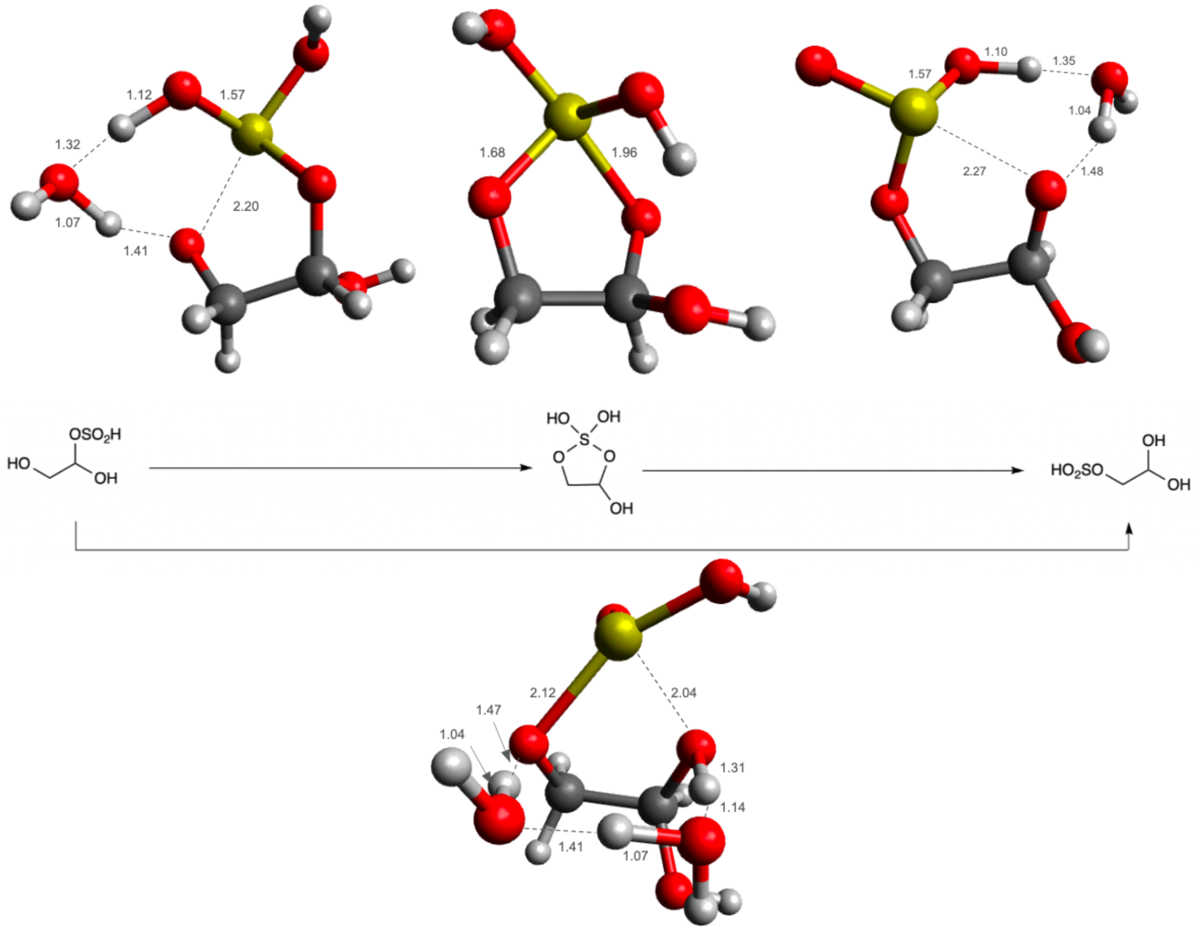

Figure S2. Structures of the transition states and intermediate for the intramolecular hopping of the bisulfite group in glycolaldehyde.

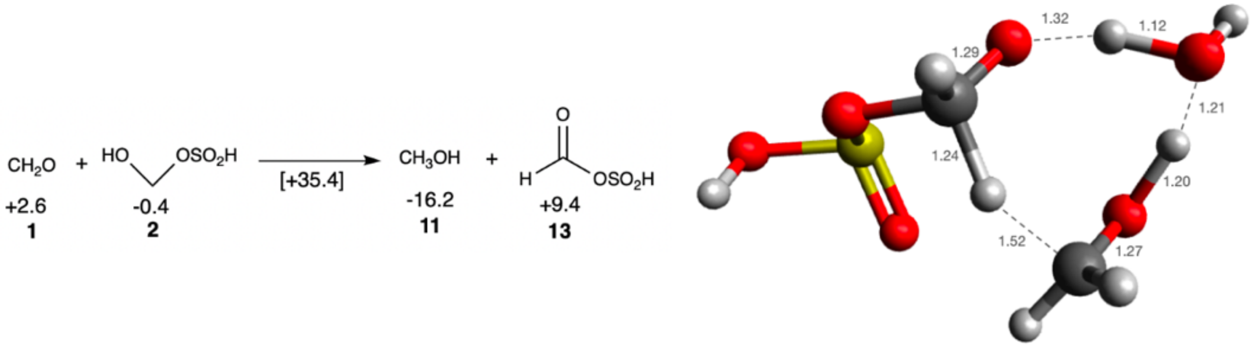

Figure S3. Transition state of the Cannizzaro addition of formaldehyde and the formaldehyde bisulfite adduct to form methanol and the bisulfite acid.

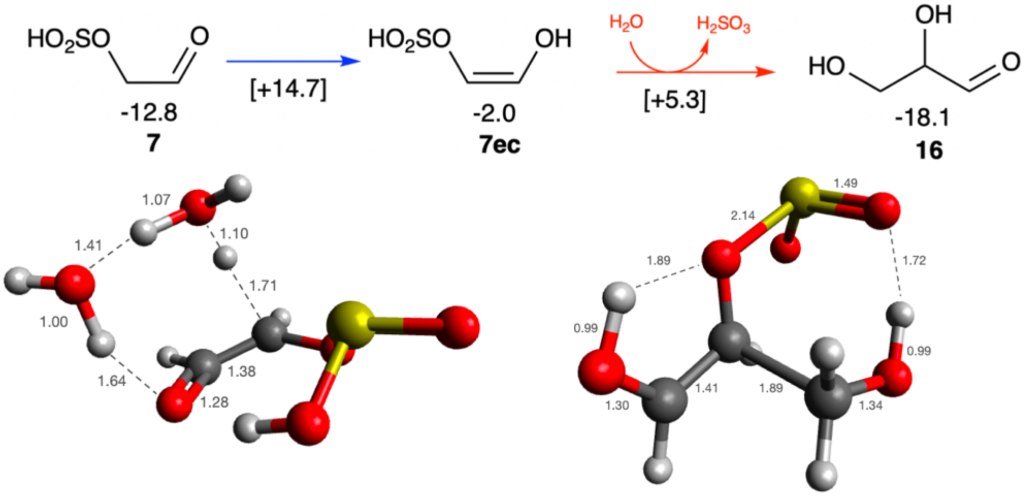

Figure S4. Transition states for enolization of glycolaldehyde-bisulfite followed by aldol addition with formaldehyde to form glyceraldehyde (with concomitant SO_2 removal).

Part 3: Free energy comparison of open tetroses and ring structures

Apart from retro-aldol and aldol addition reactions, C_4 aldoses can undergo ring closure. As a baseline, we calculated the free energy changes for non-sulfurous erythrose and threose into their α- and β- furanoses. The open and closed conformers were within 1 kcal for both diastereomers, and barriers were around +15 kcal, so both isomers are expected to be present at room temperature. We also calculated the free energy for furanoses formed by **26** and **27** with bisulfite on carbons 2 and 3 respectively. Both diastereomers of **27** were about 1.5-3 kcal more stable than their non-sulfur counterparts and preferably form the α-anomer. Though this difference is within our computational error, it may suggest that sulfite enables tetroses to be sequestered as stable rings, buffering product distribution without permanently depleting reactants.

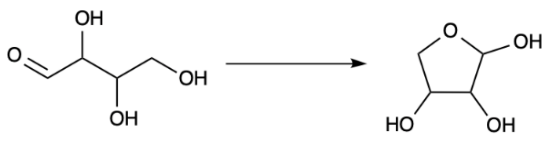

29 **ery** -23.5 **thr** -24.2

Diastereomer	β-furanose G_{rel}	α-furanose G_{rel}
Erythrose	-23.4	-24.9
Threose	-24.1	-25.4

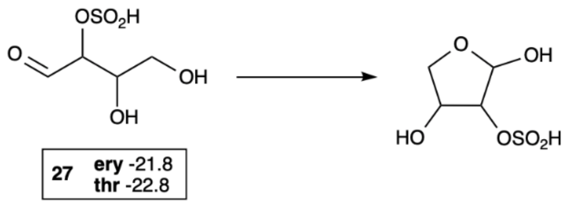

27 **ery** -21.8 **thr** -22.8

Diastereomer	β-furanose G_{rel}	α-furanose G_{rel}
Erythrose	-24.1	-24.8
Threose	-23.3	-24.7

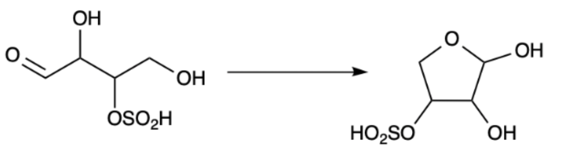

26 **ery** -23.6 **thr** -24.5

Diastereomer	β-furanose G_{rel}	α-furanose G_{rel}
Erythrose	-24.4	-25.9
Threose	-23.7	-19.8

Figure S5. Relative free energies of open C_4 tetroses versus their furanose anomers. (Energies are in kcal/mol)

Part 4: XYZ coordinates for transition states

1 → 2

C	-1.334856490	-1.423201249	-0.323899748
O	-2.214900287	-0.703490023	0.182957861
O	0.484440105	-1.145802973	0.700823168
S	1.395117768	0.035391109	0.405907632
O	0.626064192	1.350033964	0.447161619
O	1.578942191	-0.068798114	-1.299419532
H	-1.276959009	-2.473170811	-0.036325357
H	-0.798342806	-1.116581209	-1.224832777
H	-0.627922834	1.538800910	-0.017518878
H	2.116729845	-0.852759563	-1.477483837
O	-1.675745984	1.586818185	-0.322637112
H	-2.134751769	2.215886676	0.245438324
H	-2.043157976	0.534593648	-0.117132876

1 → 3

C	-0.983076855	-1.521467589	-0.437767669
O	-1.885717383	-1.118127076	0.335998113
O	-2.191492578	1.243060555	0.071609340
S	0.894476629	-0.022260705	-0.165788967
O	0.248156345	1.345020632	0.106980583
O	1.966157444	0.048064903	-1.167410410
O	1.565713209	-0.385335817	1.316947947
H	-0.436268291	-2.432984531	-0.184549884
H	-0.961498445	-1.226531904	-1.494057629
H	-2.222555610	0.110821589	0.141568337
H	-2.570630684	1.586792968	-0.744629865
H	-1.114800929	1.406736970	0.092115253
H	1.942527146	0.433552303	1.677874144

4 → 5

C	2.043506978	-0.811782832	-0.098696225
C	1.174301595	0.344012570	0.341408491
O	1.137687559	1.383190466	-0.373301118
O	3.392542434	-0.470154124	0.203347811
S	-1.053327148	-0.662982007	0.135373688
O	-1.986871450	0.545646802	-0.031843191
O	-1.561934427	-1.645825482	1.102128882
O	-1.105430687	-1.337987741	-1.394294664
O	-0.803679439	2.680558815	0.140385183
H	1.883502092	-1.002580396	-1.164286676
H	1.807018285	-1.709249613	0.472207450
H	0.958165100	0.414145809	1.415326918
H	3.647154498	0.248370375	-0.386079359
H	-1.430419709	1.800563164	0.074970159

H	-2.016464369	-1.254317696	-1.719423402
H	0.220328251	2.200526734	-0.043597804
H	-0.889358524	3.088677143	1.008827650

4 → **6** mechanism **(i)**

O	-1.530844074	-0.185424770	-1.560758872
C	-1.082357184	-1.164301336	-0.933226248
C	-1.740528019	-1.731377098	0.318036558
O	-1.960777039	-0.759693639	1.334355802
S	1.896980744	-0.346610914	-0.360307328
O	2.285132040	1.090921199	-0.418869133
O	0.447260746	-0.378812004	0.270800095
O	2.808599159	-0.989210365	0.904518962
O	-2.495973808	1.490756038	-0.104761543
O	-0.220511332	2.339315484	0.617287763
H	-0.417756435	-1.849522814	-1.471390323
H	-1.128889682	-2.554869529	0.693139166
H	-2.721032704	-2.125132737	0.028375588
H	-1.063757146	-0.495917934	1.596904415
H	0.221743041	1.463141508	0.593464074
H	2.943194319	-0.272396337	1.546351980
H	-2.126564058	0.726113922	-0.849215787
H	-2.663947720	0.900804852	0.661302143
H	-1.637390097	1.998691167	0.151149901
H	0.370610953	2.894036657	0.094454283

4 → **6** mechanism **(ii)**

C	-1.377152362	0.101571216	0.863306672
C	-1.930486586	0.647023461	-0.437104740
O	-1.101798814	-1.113928413	1.010114350
O	-3.328310504	0.369510709	-0.434782493
O	0.387546288	1.218239795	0.722063515
S	1.724240903	0.794457952	0.129533625
O	2.143128112	-0.574867365	0.588502518
O	1.233353922	0.316596116	-1.518733826
H	-1.630223140	0.663718639	1.763756742
H	-1.412998169	0.206326890	-1.294705083
H	-1.820093543	1.728013214	-0.466017411
H	-3.445395478	-0.585476328	-0.483126834
H	1.247318495	-1.737088373	0.023406250
H	1.057330801	1.118493992	-2.027750310
O	0.458191137	-2.134464021	-0.492185773
H	0.520290103	-1.619237926	-1.317594469
H	-0.426374429	-1.681807307	0.150037612

4 → **7**

C	1.334650585	-1.205129840	0.040991390
C	2.536633284	-0.284268684	0.127929910

O	2.534900715	0.876500711	-0.181033198
O	0.232909923	-0.634422671	-0.615991030
O	-1.927153473	-1.659692925	-0.167572538
S	-0.549183651	1.012603841	0.271604236
O	-1.400265298	0.190586683	1.257743597
O	-1.343853242	1.469471403	-0.868395514
H	1.100966242	-1.527610237	1.071692142
H	1.651820915	-2.108015201	-0.500079824
H	3.448864109	-0.778546575	0.531778582
H	-0.946350827	-1.386863185	-0.540318526
H	-2.586763788	-1.445237873	-0.840702218
H	-1.908340060	-0.833624482	0.611420097

6 → 9

C	1.716382948	1.101581605	0.049553308
C	0.308630568	1.658813512	-0.062894963
O	1.633143405	-0.358393896	0.069327317
O	-0.463586865	0.952309257	0.845768329
O	2.478736882	1.464979682	-1.046464495
S	0.340411761	-1.121393127	0.794312431
O	-0.617992044	-1.478128742	-0.361072722
O	1.159434413	-2.577518316	0.811261228
H	2.182189422	1.393465560	0.997463979
H	-0.009539891	1.535523853	-1.115694264
H	0.342843996	2.738386508	0.148667802
H	-1.855803652	1.294399998	0.883178929
H	3.387508656	1.184164032	-0.897281362
H	1.312564475	-2.860113113	-0.104449048
O	-2.892714995	1.355652387	0.710707143
O	-2.603275013	-0.321606843	-1.053216751
H	-3.131287373	2.273742396	0.558739865
H	-2.855260124	0.446987615	-0.356659691
H	-1.625222509	-0.837250256	-0.673922647
H	-2.431415142	0.077760261	-1.912814020

9 → 8

O	-1.621245528	-0.001846774	1.112676032
O	-0.153291612	-0.510639866	-0.895906512
C	-1.894102823	-1.240859452	0.495662590
C	-0.662731226	-1.653109503	-0.355694700
S	-0.797574991	1.291991163	0.313950552
H	-2.056513344	-1.971592374	1.286581361
H	-2.775709291	-1.144151426	-0.144572444
H	-1.010674228	-2.336351758	-1.149764143
O	0.233880444	-2.326575422	0.509095354
O	-1.643278212	1.513608533	-1.049136155
H	-1.271384626	0.830380387	-1.651975630
O	0.666731684	1.770418437	-0.295432533

H	1.425095184	1.547337745	0.331073854
H	1.115677586	-2.277023176	0.113337106
O	2.433392339	-0.996480548	-0.825438400
O	2.693554233	0.896066086	1.088674410
H	1.512523940	-0.675169191	-1.030713760
H	2.901882655	-1.076903820	-1.660783422
H	2.831581103	0.138840956	0.476294637
H	3.515200944	1.395064059	1.104894275

8 → 7

C	0.547568942	-1.348358821	0.425224722
C	-0.747269088	-0.528513793	0.375893599
O	-1.734398048	-1.475151465	-0.420701623
O	-0.665208271	0.641413635	-0.222349336
O	1.575653178	-0.619597638	1.096381681
S	2.663482834	0.267343115	0.095125666
O	2.735228429	-0.478140702	-1.173808616
O	1.754497851	1.589733334	-0.118019159
H	0.865142116	-1.589111598	-0.596350892
H	0.395808913	-2.275823632	0.980134527
H	-1.262467408	-0.533553078	1.351446276
H	-1.450902638	-1.402563328	-1.344493602
H	-1.871547425	1.432444553	-0.058546850
H	0.793504967	1.317773156	-0.280838456
O	-2.852259947	1.795862285	0.056351851
O	-3.879669868	-0.392457573	-0.194198594
H	-2.891966829	2.319384455	0.861867241
H	-3.559019298	0.638179340	-0.025128389
H	-2.887107855	-0.981863622	-0.315564114
H	-4.410383581	-0.721926517	0.538534056

6 → 8

C	0.596817278	1.305583235	-1.190483671
C	0.675762993	1.469761230	0.365007826
O	0.073801725	0.062856597	-1.513954888
O	-0.038436416	0.423571310	0.999783483
O	2.043438339	1.412966582	0.768737954
S	-1.512014269	-0.355153190	-0.172275613
H	1.617320773	1.433758786	-1.572855217
H	-0.028936597	2.112130059	-1.592145917
H	0.246782215	2.421863392	0.687465048
H	0.982347075	-1.093710966	-1.477202692
H	2.111664800	1.830792904	1.634166679
O	-2.278582190	0.853338646	-0.475593465
O	-2.133102044	-0.888503743	1.306278165
H	-2.300110942	-0.134900026	1.893986778
O	1.587682636	-1.906167557	-1.226574967
O	1.630981561	-1.345232332	1.165921520

H	1.183618086	-2.702253131	-1.583332456
H	1.638143691	-1.729742503	0.167583555
H	0.804308489	-0.563968654	1.160223639
H	2.419815744	-0.784036697	1.220205270

1+10 → 11+12

C	0.783631322	-1.544184725	0.282970772
C	-1.206951823	0.171799877	0.367971803
O	1.553010777	-0.905086381	-0.534452789
O	1.642320634	1.348389475	0.292569574
O	-0.631618555	1.025165951	-0.412566989
O	-2.425509624	-0.284944744	-0.019539923
H	0.434672090	-2.538017635	-0.037672461
H	0.999575944	-1.498195340	1.366957957
H	-1.176656465	0.305391231	1.455697040
H	-0.386063902	-0.956363614	0.353764854
H	2.177423904	1.960450868	-0.221185960
H	1.791769283	0.300952141	-0.084586308
H	0.426369128	1.320468847	-0.069172864
H	-2.486583035	-0.141168998	-0.974690789

1+2 → 11+13

C	-1.608409880	1.417561256	0.883110310
O	-2.105389768	1.322547695	-0.280188241
C	-0.361508982	-0.939747481	0.750875843
O	-0.912331043	-1.264768439	-0.372414950
O	1.059723731	-0.879876801	0.746782536
S	1.718404120	-0.000108349	-0.569770460
O	1.339653521	1.395077809	-0.321107690
O	3.251805783	-0.256846678	-0.017059597
H	-0.808095504	2.143806462	1.034593771
H	-2.196575768	1.115121909	1.759044858
H	-0.620039674	-1.500072350	1.665121587
H	-0.697424471	0.208277358	1.069227931
H	-2.224598976	-1.155335141	-0.420371274
H	3.293782993	-0.027722038	0.926204152
O	-3.266372578	-0.751846721	-0.414686245
H	-2.845147588	0.382736406	-0.387069510
H	-3.708754666	-0.956548026	-1.244768358

4+10 → 14+12

C	-1.544189015	1.317371818	0.195159096
C	-0.714643379	0.797561083	-0.994938913
C	1.503681714	0.146988574	0.326241829
O	-0.962697584	-0.393593707	-1.465585184
O	-1.309632968	0.623537003	1.411543867
O	1.479706287	-1.074915210	-0.090562933
O	2.638698880	0.798565673	0.008162882

O	-0.823155883	-1.903441674	0.392738022
H	-2.602798149	1.269313615	-0.100940726
H	-1.294609249	2.366241787	0.383198244
H	-0.572652496	1.573561973	-1.768703327
H	1.013462148	0.407305345	1.266406696
H	0.445190517	0.814967812	-0.472560383
H	-1.433718466	-0.329637730	1.258996177
H	0.597272399	-1.555088571	0.192677128
H	2.655124564	1.647730132	0.463936386
H	-1.123218317	-2.811346070	0.298379233
H	-1.027395942	-1.289896948	-0.533876617

7+10 → 15+12

C	0.541646931	-1.405027541	-0.556225024
C	-0.379923071	-0.178101613	-0.564339494
C	-2.759849285	-0.512241734	0.323580527
O	-0.238629416	0.692562316	0.397526323
O	-3.456389469	0.352633437	-0.348622296
O	-3.387823470	-1.687708883	0.457567062
O	1.882227189	-1.055533419	-0.888536907
S	2.918189840	-0.230130638	0.237166599
O	3.074218525	1.108399096	-0.330467130
O	1.917725220	-0.172924166	1.524287132
H	0.227448979	-2.116937873	-1.324270061
H	0.496565122	-1.888545346	0.426087914
H	-0.558049463	0.227934003	-1.574587745
H	-2.122208793	-0.142957181	1.145816440
H	-1.537743011	-0.752285932	-0.420599481
H	-3.005165113	1.271312650	-0.296321684
H	-2.875779764	-2.263861501	1.037469544
H	1.064653173	0.306096887	1.263481984
O	-2.056709830	2.470207122	-0.198420893
H	-1.211608451	2.012238135	0.071910220
H	-2.200432996	3.205203398	0.403996747

4+2 → 14+13

C	-1.396496304	-1.697936244	-0.577714845
C	-2.086249626	-0.465766314	-1.124852273
O	-1.278865328	-1.697742168	0.807404043
O	-2.841203946	0.275481837	-0.424592960
O	-0.064950780	1.825273171	0.120808683
C	0.109751872	1.251357729	-1.007178165
O	1.318721237	0.357859188	-1.102737887
S	2.090705035	0.138649967	0.356382995
O	1.330922526	-0.916228181	1.065951610
O	3.397440777	-0.585296559	-0.349200823
H	-0.444451759	-1.841944538	-1.102856142
H	-2.058540292	-2.526023301	-0.885300788

H	-2.285383429	-0.460986709	-2.202955810
H	-0.368857561	-1.433717706	1.039013722
H	-1.101670576	1.398135639	1.173894246
H	0.195690444	1.874146123	-1.914607333
H	-0.741400465	0.465967325	-1.292391376
H	3.088337682	-1.286501507	-0.945288514
O	-1.936492000	1.070557306	1.664191976
H	-2.501589045	0.491543755	0.636589256
H	-2.414048795	1.860701293	1.941089886

7+2 → 15+13

C	1.622987231	1.611632191	-0.829593657
C	0.540768572	1.365077228	0.226778079
C	-0.718220871	-0.838284362	-0.142139424
O	0.877171708	0.905228275	1.397294939
O	2.240037291	0.434886481	-1.352227648
S	3.201698467	-0.611525785	-0.385398467
O	2.293557655	-1.724171466	-0.073852835
O	3.388228980	0.317160206	0.934895595
O	-0.651150476	-0.955906412	1.160179078
O	-1.951679264	-0.801218923	-0.712631489
S	-3.296984046	-0.194553392	0.258588361
O	-4.427403096	-0.540098263	-0.589462989
O	-2.961629381	1.412842534	0.035010568
H	2.371288851	2.281364361	-0.389332217
H	1.179390896	2.104562509	-1.697079798
H	-0.255312600	2.128223280	0.215059282
H	-0.016616518	-1.402707207	-0.763462367
H	-0.155633151	0.395728557	-0.360877062
H	2.485893577	0.493813425	1.346303206
H	0.068944442	-0.270309115	1.509754161
H	-3.245265690	1.669524741	-0.859901007

7 → 7ec

C	-0.205296338	-1.624610696	-0.544230613
C	-1.239117700	-1.420141953	0.354271193
O	-1.280538645	-0.564097881	1.310200151
O	1.113173190	-1.206924252	-0.307926449
S	1.416981561	0.473654592	-0.095822724
O	2.860168159	0.613244847	-0.223216296
O	1.076099697	0.584536530	1.484841862
O	-1.375886502	0.597949770	-1.761241334
O	-2.634750706	1.353006045	0.228423481
H	-1.011519441	-0.367724707	-1.379601280
H	-0.244102891	-2.468439529	-1.224338980
H	-2.163568176	-1.967550736	0.107192109
H	0.173760969	0.154945021	1.614627029
H	-1.991733551	0.987674408	-0.974861319

H	-0.590264335	1.165892987	-1.828973647
H	-2.264532567	0.672166113	0.867012112
H	-3.594690165	1.340407912	0.288415100

7ec → 16

C	-0.924681969	-0.201324394	0.497556351
C	-2.320218182	-0.386927892	0.523914833
O	-2.849249001	-1.030000577	-0.477524263
O	-0.307714421	-0.913085394	-0.440744203
S	1.820822847	-0.711787037	-0.224049387
O	1.867017772	-0.718824618	1.245076680
O	0.011438589	2.217211622	0.239586237
C	-1.098049076	1.567557565	-0.144526218
H	-0.403699151	0.015351842	1.435112224
H	-3.011318529	0.049170046	1.234356459
H	-2.060589112	-1.386783455	-0.967269711
H	-1.163575295	1.277459409	-1.195741847
H	-1.979387360	2.081503884	0.233380787
O	1.909038551	0.649618453	-0.822123649
H	0.808667450	1.798109616	-0.175324195

7ec → 17

C	0.001218331	0.946486815	1.198770252
C	0.804747070	-0.161316176	1.252452373
O	0.676989115	-1.241457358	0.510402981
O	-1.067010693	1.159260211	0.391408331
S	-1.847367460	-0.136923368	-0.588319356
O	-3.194314985	0.384102132	-0.739074882
O	-1.915562637	-1.311101723	0.533819546
H	0.140345362	1.743771562	1.921418629
H	1.622890097	-0.113829158	1.976982356
H	1.874795135	-1.497476152	-0.109522244
H	-0.971120770	-1.624069794	0.607583300
C	1.771539021	1.418348934	-0.508393251
O	2.837376082	-1.398021485	-0.604440762
H	1.587177941	2.465571137	-0.269654803
H	0.988415730	0.868947413	-1.032766214
H	2.987774252	-0.201191789	-0.644457664
H	2.823180165	-1.825440587	-1.466874690
O	2.958723478	0.996682448	-0.484978410

7 → 7et

C	0.414489243	-0.201136884	-0.192415604
C	0.786284314	0.888883368	0.627224634
O	1.853034344	1.552477757	0.525258008
O	-0.829361375	-0.823442919	0.048870893
S	-2.241543831	0.006506194	-0.525329313
O	-3.201346673	-0.869609732	0.493836534

O	-2.118217838	1.393059647	-0.066644029
O	2.581122707	-1.654140298	0.317461326
O	3.697256104	0.303340503	-0.500719847
H	1.573047171	-1.184627447	0.207042948
H	0.698108874	-0.158355802	-1.245921364
H	0.129074655	1.107479054	1.480126237
H	-2.741157801	-0.966366426	1.344605656
H	3.243755127	-0.792496972	-0.142549221
H	2.606443008	-2.478664219	-0.181408055
H	3.781853191	0.408258081	-1.453584708
H	2.918858957	0.957216172	-0.141748773

7et → 17

C	-0.924740664	1.546358659	-0.029724057
C	-0.207724178	0.588339632	0.692025289
O	-2.175876027	1.605409845	-0.232604902
O	1.161295899	0.916368675	0.905275546
S	2.348815571	0.280755045	-0.093511484
O	1.813630695	0.116835182	-1.443677013
O	2.352891539	-1.308403634	0.479225585
H	-0.309962062	2.306895980	-0.529573846
H	-0.628376527	0.024831563	1.513139472
H	-2.958575340	0.600525615	0.046743116
H	2.613948193	-1.302422553	1.412104544
O	-1.714709985	-1.850167758	0.143555152
C	-0.784592967	-1.322316825	-0.529048137
O	-3.562600993	-0.336080598	0.167307701
H	0.214345527	-1.744253325	-0.457145727
H	-1.004576014	-0.698821144	-1.397810422
H	-4.215812480	-0.393567396	-0.537298236
H	-2.747045996	-1.176888986	0.107343881

16 → 17

C	1.668803228	-1.451769532	-0.135377979
C	1.265918802	0.012071853	-0.004753030
C	2.417735207	0.815859410	0.606417398
O	0.101716922	0.188994883	0.785976606
O	0.684118815	-2.172077763	-0.907437176
O	3.578990998	0.506446181	0.521191562
H	1.769529577	-1.903500241	0.858519218
H	2.641688167	-1.484000181	-0.628744454
H	1.103153565	0.420274159	-1.014451542
H	2.084927368	1.724850522	1.144331949
H	-0.787970089	-1.193920283	1.092798933
H	1.112606736	-2.939670530	-1.296987111
O	-3.306371468	-1.012450258	0.055906242
O	-2.024999734	0.931099627	-0.649394615
S	-1.005986647	1.681168849	0.206175092

O	-0.074028438	2.424562494	-0.654581086
H	-2.823779926	-0.097392422	-0.257716315
H	-3.710472063	-1.434862690	-0.710120554
O	-1.294814488	-2.039048932	0.907072376
H	-0.746387473	-2.388492567	0.170715617
H	-2.418371457	-1.604327969	0.452165474

4ec → 16

C	1.453931109	0.463196643	0.410643800
C	0.422103197	1.196732302	-0.234380657
C	-1.030990740	0.850790626	0.948051545
O	-1.774714781	0.013263725	0.279733727
O	-0.062389295	0.697684203	-1.427828947
O	1.634193770	-0.789808767	0.360848286
O	-0.376445470	-1.997309076	-0.187631987
H	2.110174024	1.007919328	1.098494591
H	0.571391770	2.276888972	-0.211291090
H	-0.577056562	0.500992331	1.885775000
H	-1.408368892	1.877488111	1.038737974
H	-0.994533325	0.441876025	-1.222907341
H	0.729100268	-1.390174327	-0.033093586
H	-0.520262595	-2.220282208	-1.113818653
H	-1.079736878	-1.247626786	0.062465710

17 → 17ec

C	1.557813831	1.855022974	0.838921113
C	0.608047820	0.697204565	0.749452959
C	0.919620077	-0.552914202	1.265629492
O	1.967556809	2.310865485	-0.469034768
O	-0.674612442	1.124852068	0.392617493
O	0.209745065	-1.621975300	1.247894822
S	-1.759420489	0.044710714	-0.470186803
O	-2.869438878	0.921675663	-0.815149659
O	-2.255491632	-0.852074642	0.789637986
H	2.474472989	1.556029512	1.349580538
H	1.103427504	2.680470838	1.397003869
H	1.215009112	0.020520529	-0.587852265
H	1.970487457	-0.641441173	1.589169076
H	1.199619560	2.736210227	-0.867253103
H	-1.424928085	-1.317218349	1.106836440
O	1.544656048	-0.401164480	-1.569114011
O	1.462101253	-2.774976114	-0.782997459
H	2.362713787	0.062106534	-1.798792284
H	1.661556722	-1.418840466	-1.380442795
H	2.181871541	-3.368365601	-0.550192881
H	0.951004877	-2.580769008	0.052073523

17 → 17et

C	0.788286783	1.691927790	0.441819851
C	0.307491085	0.285018713	0.726045860
C	1.069487519	-0.626308595	1.485246665
O	-1.090746174	0.214019687	1.047507513
O	0.319170557	2.229805006	-0.794812134
O	2.325309024	-0.756295290	1.484150318
S	-2.130263461	0.046500150	-0.261432683
O	-3.434422025	-0.109469545	0.377501602
O	-1.642531738	-1.492450624	-0.780201287
H	1.884597009	1.701635869	0.500078529
H	0.404512357	2.370331925	1.208335410
H	0.677153445	-0.342250774	-0.656120437
H	0.480326242	-1.334422189	2.089962124
H	0.590316173	1.632531365	-1.501490179
H	-1.980170710	-2.141474194	-0.139977914
O	1.164791974	-0.535667130	-1.650029729
O	3.375000291	-0.608687144	-0.732074677
H	2.316419596	-0.617231818	-1.327687506
H	0.757458385	-1.326489078	-2.025574761
H	3.039833911	-0.593562452	0.279449649
H	3.916646817	0.166560020	-0.912576063

17 → 18

C	1.167730492	-0.625285378	-1.231934631
C	1.426128950	0.626903518	-0.304661302
C	2.666261814	0.381780845	0.528107430
O	0.300081020	0.837976574	0.529659302
O	3.774537343	0.671453443	0.161971758
O	-0.187816712	-0.780844574	-1.424557493
S	-1.421729435	0.824841223	-0.617325252
O	-2.403516801	-0.160964548	-0.174178899
O	-1.863577613	2.079959656	0.443942140
H	1.605627285	-1.513624253	-0.738380977
H	1.730572832	-0.462634090	-2.161445339
H	1.585275132	1.521277333	-0.913266870
H	2.482950378	-0.139686628	1.493655791
H	-0.603814182	-2.042855030	-0.634432361
H	-1.099583661	2.664119204	0.552247826
O	-0.452636372	-1.010198324	1.910428196
O	-0.725451109	-2.714631450	0.132631712
H	-0.017099655	-0.121869761	1.296690564
H	-1.358042936	-0.765051008	2.150220067
H	-0.125036508	-3.451996385	-0.006122384
H	-0.546643013	-1.824591382	1.236968106

18 → 18ec

C	0.384132827	-1.747912452	0.088708563
C	-0.849907651	-0.863036884	-0.036099839

C	-2.060562215	-1.254033487	0.588886988
O	-0.980150081	-0.314976882	-1.334477666
O	-3.197071119	-0.740871981	0.388978282
O	1.520295640	-1.103941700	-0.501095056
S	2.235189161	0.185490274	0.402931074
O	3.607105150	0.208193986	-0.085767913
O	1.471312084	1.427166246	-0.338495579
H	0.272015552	-2.666662581	-0.491784320
H	0.568495324	-2.011042629	1.137006111
H	-0.764008146	0.234197136	0.718909874
H	-1.980988302	-1.964924185	1.421683274
H	-0.085746187	-0.050952822	-1.599303586
H	0.635961055	1.651576138	0.185385431
O	-0.802241521	1.569589366	0.907917887
O	-2.891489227	1.473300983	-0.517527357
H	-0.829031374	1.828781217	1.835236201
H	-2.088525580	1.702523425	0.103796932
H	-3.130021055	0.376629805	-0.165428743
H	-2.459846444	1.320077085	-1.372856021

18 → 18et

C	0.068925316	0.626160010	1.232422754
C	0.839102098	1.285038251	0.147218106
C	2.238606344	1.226442597	0.169029041
O	2.934869159	0.387341610	0.815346776
O	0.253595983	2.409960891	-0.456676310
O	-0.867595689	-0.460925467	0.739230588
O	0.928556116	-1.140932756	-1.152067146
O	3.121257496	-1.871595501	-0.313480511
H	-0.544356696	1.342548105	1.785255699
H	0.733116358	0.086731662	1.904568560
H	0.987169280	-0.104282364	-0.853048984
H	2.732291198	1.963570065	-0.489494288
H	-0.524587750	2.114314641	-0.945031586
H	0.197927341	-1.441017358	-0.575107600
H	1.860845693	-1.584974761	-0.822373835
H	3.176597209	-2.579870443	0.335524698
H	3.254618435	-0.997313245	0.187101999
S	-2.428233686	-0.008668058	0.388199955
O	-3.060217892	-1.266370092	-0.010972815
O	-2.158206592	0.841733716	-1.051947634
H	-2.201685673	0.202115240	-1.783466815

18ec →19

C	0.315039614	-1.886098579	-0.124890429
C	-0.876125063	-1.043161997	0.315714623
C	-2.030660228	-1.098649741	-0.448869344
O	-0.784501079	-0.323452463	1.376090169

O	-3.222670475	-0.577549932	0.084738616
O	1.593103059	-1.353222635	0.263866187
S	2.041513260	0.135382630	-0.403308398
O	3.493501009	0.171321921	-0.318272007
O	1.456119849	1.053028669	0.873904231
H	0.279518498	-2.076622864	-1.202742273
H	0.273347483	-2.847491852	0.391846965
H	-2.181397560	-1.871289209	-1.196598605
H	-1.183779658	1.333276833	1.116305820
H	-3.070743775	-0.479375690	1.035908519
H	0.836824165	0.463732548	1.379987347
O	-1.123996114	2.204252760	0.634638323
H	-0.167513909	2.380438792	0.652060467
H	-1.503878866	1.825107733	-0.602129472
O	-1.817538934	1.326027900	-1.537638111
H	-2.774611220	1.440872090	-1.623981783
H	-1.698120971	0.271123200	-1.272993479

17ec → **19e** (ring proxy)

C	-0.719197616	-1.575747140	-0.106064497
C	-1.143439991	-0.335849462	0.177744670
C	-2.458515549	0.171540473	0.633836074
O	-0.140215402	0.662493724	0.012772765
O	0.531301288	-1.727337557	-0.513248360
S	1.373027909	0.160695520	-0.421259472
O	2.069930400	-0.440337996	0.942900305
H	-1.347445495	-2.456441083	0.000352605
H	-3.056134335	-0.700441091	0.935753224
H	1.800021811	-1.378852456	0.944328741
O	1.985774902	1.675929308	-0.150178107
H	1.734455233	2.006918611	0.727054390
O	-3.146028204	0.975287908	-0.325556662
H	-2.343209646	0.814664878	1.510227763
H	-3.179863248	0.471969390	-1.146063144

17ec → **21**

C	-1.894886231	-1.941803218	-0.096593713
C	-0.784610973	-0.986751801	-0.467257662
C	-1.018347920	0.196825814	-1.101528407
C	-1.290024680	1.159869081	1.093226323
O	-2.253162670	0.497646071	-1.629980679
O	-1.914978610	-2.152540613	1.311862269
O	0.394442986	-1.390756526	-0.017983081
S	2.060986762	-0.477748094	-0.469310646
O	2.990520970	-1.433382431	0.116677960
O	1.909946827	0.751849153	0.446442729
O	0.910697197	2.916868201	0.115295048
H	-1.741326086	-2.884010870	-0.640231375

H	-2.861699813	-1.529636578	-0.378967556
H	-0.201597183	0.846927370	-1.400311111
H	-2.196800430	0.595468317	1.320414578
H	-0.338258667	0.737008515	1.428004488
H	-2.435132887	1.429015829	-1.446029509
H	-1.024071863	-2.431859061	1.556588675
H	1.417645880	1.916006684	0.199107250
H	-0.135485385	2.783197563	0.423232555
H	1.359681498	3.563226249	0.671751763
O	-1.379632854	2.356714988	0.729403169

17ec → 21

C	-2.384435770	0.494809040	-0.760886415
C	-0.936654316	0.022498889	-0.579440461
C	-0.656960585	-1.310131223	-0.920217000
O	-2.879854745	1.232995616	0.348916105
O	0.489719165	-1.898411459	-1.053667260
O	0.179464790	-1.383021414	1.709941404
C	-0.952823936	-0.816231777	1.329716425
O	-0.091787801	1.056908536	-0.558480646
S	1.786639658	1.050026323	-0.109390435
O	1.731963439	0.666158037	1.333502876
O	2.252997551	-0.084827543	-0.973349301
H	-3.037865036	-0.348939963	-1.007122211
H	-2.369412334	1.174793050	-1.615882218
H	-1.501408729	-1.985831240	-1.020724594
H	-3.455363588	0.665543541	0.867308967
H	1.275784552	-1.249041213	-1.039678827
H	-1.132000174	0.216979351	1.620195911
H	-1.807053570	-1.484712441	1.399381218
H	0.891541009	-0.684885595	1.750512591

18et → 22

C	1.038955038	-1.402003495	-0.223012073
C	-0.255392635	-1.129645265	0.491533592
C	-0.708210112	0.151933869	0.787100335
C	-1.693859728	-1.177219036	-1.090092339
O	-0.764002973	-2.177867597	1.240911746
O	-0.308641647	1.262357360	0.272359696
O	2.189339272	-0.945675766	0.529247259
S	3.116200914	0.321548426	-0.118343309
O	2.834192189	0.341748397	-1.564822495
O	2.363309068	1.595268230	0.574670590
O	-2.830628736	-1.441633123	-0.578143556
O	-3.950480784	0.719735499	-0.227731697
H	1.061174576	-0.935200625	-1.213598658
H	1.148179683	-2.481275696	-0.332981020
H	-1.588999086	0.183913727	1.442803668

H	-1.494106556	-0.196471793	-1.537783828
H	-1.121567753	-2.006750706	-1.519430123
H	-1.703986529	-2.277368026	1.009666444
H	-1.385344617	2.064580757	0.224581457
H	1.396162807	1.599971742	0.358238081
H	-4.565536692	0.880939375	-0.950025544
H	-3.504874418	-0.264890366	-0.360519637
O	-2.386391207	2.515096549	0.154729003
H	-2.581456304	3.025731127	0.947381432
H	-3.172115777	1.670070403	-0.032644351

18ec → 22

C	1.040583018	0.209392519	-0.950826784
C	-0.125954573	0.019586655	-0.047662209
C	-1.126449863	-0.914300845	-0.273137362
C	-1.633515384	1.551714911	-0.761103178
O	2.151917174	-0.681342928	-0.646971290
O	0.060430165	0.488954049	1.225343765
O	-1.969130115	-1.199373922	0.648795555
O	-2.476752288	1.688673903	0.169936483
O	-4.200167804	-1.716932552	-0.104905787
H	1.397753041	1.243836458	-0.923170020
H	0.769933165	-0.040584449	-1.977493725
H	-1.234495610	-1.335070713	-1.279122609
H	-0.755626993	2.201207381	-0.762435544
H	-1.938125272	1.153320006	-1.737493934
H	-0.727651792	0.224837545	1.725814364
H	-4.709267642	-2.193327368	0.558714589
H	-4.513876073	-0.546124206	-0.160100680
S	3.155199385	-0.229817973	0.629778879
O	4.097113056	-1.346708004	0.685476065
O	3.929038040	1.045540314	-0.131840264
H	4.494687485	0.676909845	-0.831611889
O	-4.633596990	0.638614244	-0.172284908
H	-5.063182055	0.974486969	-0.963895369
H	-3.640921432	1.136457849	-0.013922590
H	-3.155504716	-1.606523059	0.252197541

18et → 23

C	0.440884049	-1.471276420	1.070670983
C	-0.922309516	-0.884987537	0.807629203
C	-1.607344307	-1.172600219	-0.396758539
O	-1.273802173	0.067855876	1.605054267
O	-1.220332518	-2.289319046	-1.125996712
O	1.298867580	-1.070144432	0.004221820
S	2.154261836	0.489870620	0.338695429
O	3.523921871	0.243503921	-0.089626435
O	1.480083066	1.373941566	-0.803816275

H	0.394969655	-2.563015884	1.064850617
H	0.794076660	-1.129221413	2.048483069
H	-2.689227489	-1.094857266	-0.348795903
H	-2.108761423	0.589214187	1.327572639
H	-0.276903082	-2.204668695	-1.323188612
H	0.441989540	1.509162497	-0.720600793
C	-1.230941177	0.337305525	-1.488163364
O	-1.022531294	1.458700221	-0.863048523
O	-3.027184525	1.695106024	0.774177076
H	-2.149434318	0.257287983	-2.083489725
H	-0.365011576	-0.112984713	-1.987196730
H	-2.394987335	1.897241745	0.033838761
H	-3.913520158	1.624639845	0.409528766

18ec → 23

C	0.194875274	-1.647701541	0.358095672
C	-1.144998230	-1.024580696	0.624982081
C	-2.308193498	-1.587408841	0.171909045
C	-1.787611676	-0.046358797	-1.634382326
O	-3.518059540	-1.088130909	0.493301571
O	-1.263174718	0.074514059	1.363478235
O	0.989419266	-0.761227631	-0.428192994
S	2.651665769	-0.551301399	0.133364544
O	3.524358859	-1.015347704	-0.941355860
O	2.627005713	1.043960243	0.056098282
O	-2.374679402	1.000880635	-1.230951623
O	0.543221786	1.774992901	1.267059609
H	0.062713306	-2.602364049	-0.162944636
H	0.685468080	-1.851665276	1.320311279
H	-2.340891207	-2.534182864	-0.348536366
H	-2.389264765	-0.780550420	-2.164672374
H	-0.701055982	-0.082529104	-1.716772952
H	-3.361916971	-0.256238115	0.966678910
H	-0.426158678	0.716314041	1.384612478
H	1.807013897	1.397061026	0.594854849
H	0.675628940	2.282385933	2.073034252
H	-0.213988435	2.435371545	0.245797706
O	-0.832169405	2.735277286	-0.558219634
H	-1.666158856	1.814100764	-0.883426521
H	-1.253603666	3.571893817	-0.341756589

19e → 24

C	-2.174656780	-1.633397507	-0.503342533
C	-0.842226841	-0.942239503	-0.670245986
C	0.336079974	-1.451535027	-0.024896269
O	-0.744135884	0.208295293	-1.205556159
O	-3.255189316	-0.756967788	-0.378201041

O	1.555775517	-1.005461779	-0.539742768
S	2.226158944	0.558744132	-0.080347502
O	3.069739228	0.282326187	1.080962104
O	3.216716000	0.524404591	-1.409122259
O	-2.320797797	1.098714970	1.380148541
O	0.034814780	0.478544837	1.599716193
C	0.109238039	-0.836761685	1.594194837
H	-2.091945054	-2.348116635	0.327890465
H	-2.330366102	-2.229167102	-1.413047566
H	0.358697181	-2.534621088	0.100699810
H	-1.597279768	0.817584036	-1.293009555
H	-3.089736027	-0.177025203	0.398402249
H	3.813615662	-0.238313905	-1.332316357
H	-2.701473923	1.376576832	2.217402365
H	-1.252851202	0.881663893	1.527089658
H	1.034322037	-1.249126085	2.025967910
H	-0.766066090	-1.389432045	1.977326665
O	-2.457904361	2.004261045	-1.083251391
H	-2.545252509	1.897010289	-0.098171129
H	-1.964147861	2.819846085	-1.221770991

24 → **24ec**

C	-2.219212802	-0.514541631	0.256966868
C	-0.999564870	-0.449035858	-0.408902689
C	0.263576245	-1.013589551	0.225690979
C	0.360178402	-2.539650518	-0.001459470
O	-0.975074966	0.208087241	-1.538837547
O	-3.391188626	-0.132169011	-0.367073068
O	1.457975892	-0.493047239	-0.331101399
S	1.791594870	1.247136809	-0.165705915
O	1.071453514	1.597939687	1.121307218
O	1.002729984	1.726435140	-1.368810212
O	1.521166327	-3.049816033	0.606542589
H	-1.749788595	0.585374931	0.972361929
H	-2.377832995	-1.242377817	1.046953117
H	0.234485747	-0.831530751	1.306868918
H	0.338478875	-2.733025833	-1.082637916
H	-0.493887069	-3.039642987	0.460274508
H	-3.147570528	0.253882946	-1.220161968
H	-0.116467553	0.792774635	-1.634572520
H	2.255347603	-2.518864560	0.273181384
O	-1.398470557	1.606456312	1.684218066
H	-0.395050495	1.698737923	1.489151056
H	-1.838095835	2.413570755	1.390496261

24ec → **26**(thr)

C	1.742402622	1.635533387	-0.914580682
C	0.692677622	0.968002694	-0.256703870

C	-0.731165638	1.322411060	-0.544070564
C	-1.412858711	2.117279901	0.574834263
O	2.961422024	1.646419056	-0.608195357
O	0.828122025	0.444941367	1.049546642
O	-2.835372877	2.025641009	0.405497164
H	1.442043775	2.112989782	-1.864718868
O	-1.530567772	0.117596057	-0.854228910
H	-1.070168317	3.155868778	0.501522791
H	-1.108663769	1.716501918	1.544304777
H	3.478845911	0.693369454	0.482284496
H	1.768939247	0.326349019	1.271085220
H	-3.245083054	2.550639003	1.099809686
O	3.585581471	-0.025778263	1.197101974
O	2.864152179	-2.359464030	0.099055964
O	0.951011768	-1.447146881	-1.368408316
H	3.289666902	-1.577602543	0.527004671
H	4.262353461	0.263146224	1.814762709
H	1.714776263	-1.853612333	-0.827797922
H	2.530297868	-2.898822140	0.823321272
H	0.880680110	-0.349019852	-1.081601605
H	0.111455027	-1.856751709	-1.047486392
S	-1.929040325	-0.907266343	0.389406605
O	-1.307018521	-2.197526824	0.015743998
O	-3.511589161	-1.070408425	-0.072735937
H	-0.797523104	1.871455211	-1.483154643
H	-3.842817944	-0.158780874	-0.146060948

26(thr) → **4ec**+**4**

C	0.468901424	2.300165228	-0.284153416
C	0.749487397	1.385700682	0.765928524
C	1.114227183	-0.172859816	-0.143172534
C	2.593834491	-0.458713642	0.105640616
O	-0.650711620	2.399333304	-0.886134081
O	-0.334866245	1.105926805	1.565935602
O	0.305511013	-1.058998736	0.413090546
S	-1.661817915	-1.218785254	-0.408203436
O	-1.696343251	0.125357177	-1.076706007
O	-2.348767092	-1.278262608	0.880938196
O	2.972117756	-1.643275696	-0.565707304
H	1.255598432	2.938248145	-0.687772018
H	1.673306624	1.622098114	1.302171805
H	0.908632725	0.032661966	-1.201130915
H	2.765437596	-0.526955933	1.188367530
H	3.213087771	0.345642582	-0.300716289
H	-0.403185079	0.129246605	1.624068700
H	2.394497237	-2.342741805	-0.237894178
H	-1.259111208	1.597324533	-0.697540653

24 → **24et**

C	-1.652221254	1.558597983	-0.481353421
C	-0.771212588	0.531490334	-0.801984454
C	-0.648477529	-0.680077078	0.105558152
C	-1.774568858	-1.697006578	-0.188671638
O	0.002137256	0.701369149	-1.839810122
O	0.556974739	-1.405406322	-0.063931959
S	2.103503940	-0.598328681	0.277744954
O	1.687653937	0.479178350	1.275900939
O	2.339282103	0.003271833	-1.083564568
O	-1.677086540	-2.797315125	0.685382998
H	-0.563642756	2.121734255	0.278805047
O	-2.693272395	1.261948041	0.405633534
H	-0.758682376	-0.344366604	1.142068038
H	-1.698337030	-2.000286297	-1.241692342
H	-2.743269608	-1.227316783	-0.018467184
H	0.940124469	0.319671616	-1.699123327
H	-0.773696631	-3.127236895	0.604606271
O	0.371093576	2.567049674	0.957249239
H	1.005301810	1.735359317	1.072732178
H	-3.068123459	2.086184747	0.727859444
H	-1.854024856	2.300955982	-1.253932460
H	0.092778059	2.839236240	1.840168812

24et → **26**(ery)

C	2.181922795	0.795930680	-1.104830594
C	1.369133868	1.343666638	-0.109087300
C	-0.122636858	1.218525337	-0.216595938
C	-0.864591894	2.519458008	0.093937542
O	1.880962065	2.511312854	0.519411751
O	1.891781055	-0.142284193	-1.903731759
O	-0.605835764	0.167362180	0.734702105
S	-2.074934529	-0.579390596	0.430388497
O	-2.489463419	-0.255004333	-0.947289384
O	-1.466530726	-2.144144314	0.345345367
O	-2.274102796	2.357256654	0.152917051
O	1.139135018	-2.644681579	1.115948914
O	2.209157416	-0.568248781	1.696289876
H	3.201148304	1.219972661	-1.145663205
H	-0.379738365	0.855873422	-1.213579715
H	-0.567456212	3.267141989	-0.651940243
H	-0.566344871	2.892378577	1.072627797
H	1.852667519	2.387505261	1.472806796
H	1.072421523	-1.350549069	-1.630048853
H	-0.954816909	-2.215414772	-0.500361762
H	-2.577916934	1.991879543	-0.686606750
H	0.184502424	-2.563242742	1.317557563
H	1.677344279	-1.637375690	1.443574121

H	1.897139091	0.123983687	0.957026993
H	3.172274657	-0.619462797	1.657447420
O	0.643472984	-2.263701519	-1.394710051
H	1.130716783	-2.650063311	0.107648238
H	0.775915274	-2.837128870	-2.155768470

26(ery) → **4et+4**

C	-0.401199055	2.113587468	-0.496069801
C	-0.596484850	1.251620914	0.607363052
C	-0.783798715	-0.502343479	-0.196753388
C	-2.275496816	-0.795658660	-0.048758745
O	0.007936058	-1.315297722	0.442655017
S	1.980555659	-0.981785178	0.101085764
O	2.258983505	-0.215633412	1.324028055
O	1.841630189	-0.086253665	-1.116419172
O	-2.623688433	-2.033549935	-0.634474245
O	-1.627504903	1.561464791	1.492949229
O	0.541754023	2.028928887	-1.361088054
H	0.296338873	0.983327860	1.165975221
H	-0.485615896	-0.280306381	-1.232586862
H	-1.155954506	2.868220459	-0.733381511
H	-2.843510659	0.035175022	-0.499886947
H	-2.528167837	-0.864962857	1.007946378
H	1.180581830	1.212949323	-1.198324527
H	-2.391223690	-2.009602756	-1.568584714
H	-2.440093896	1.735381046	1.007608014

27(ery) → **4+7et**

C	-0.171843653	-1.217600793	-1.140884484
C	-0.151498076	-0.104630828	-0.232426235
C	1.160851414	0.900118357	-0.830382643
C	1.072457959	2.165446709	0.054452784
O	0.642577216	-2.172305269	-1.149385023
O	-1.338399016	0.675620723	-0.285161404
S	-2.597375740	0.173410716	0.743779522
O	-3.612769873	1.201924688	0.548788223
O	-3.081570807	-1.181577718	-0.101802835
O	2.307337020	0.274334062	-0.757340979
O	2.124448903	3.028546596	-0.326938008
H	0.183787641	-0.326115679	0.781632486
H	0.830396563	1.147962153	-1.855494133
H	-0.863157221	-1.157744036	-1.984818868
H	1.163215607	1.863729960	1.112805884
H	0.135601382	2.705450961	-0.078702908
H	-3.647748422	-0.894980420	-0.839523927
H	2.935103007	-0.086684116	0.528080272
H	2.888106393	2.453874146	-0.475102368
O	2.017822082	-2.617586122	0.778376626

H	1.305969411	-2.325858276	-0.233710414
H	1.589619867	-3.097758574	1.492043421
O	3.291718573	-0.571101925	1.377211110
H	2.576390774	-1.821307196	1.149726294
H	3.128365632	-0.007937741	2.138159746

27(thr) → **4+7ec**

C	0.420599241	-0.463869967	-1.759070067
C	0.201794479	-1.024018461	-0.493957586
C	1.909016544	-0.270803972	0.531438308
C	3.056579810	-1.104805880	-0.012661740
O	0.095424810	0.689674924	-2.147860983
O	1.996846719	0.984265125	0.371619384
O	4.202639341	-0.882392170	0.804514852
O	-1.141050269	2.260005236	-0.748489394
H	1.038845710	-1.066658165	-2.439411189
H	1.428342817	-0.656985839	1.441064145
H	3.249189434	-0.822711395	-1.055160932
H	2.839157667	-2.172477451	0.041489124
H	1.024177608	1.826067316	0.942746594
H	4.361131004	0.068516592	0.792083553
H	-0.597156801	1.530624137	-1.361020983
H	-1.289456750	3.060361671	-1.261923056
O	-0.723233567	-0.416485177	0.376624559
S	-2.374701703	-0.690941558	0.016572257
O	-3.062299247	-0.003437513	1.105884877
O	-2.410045039	-2.324019606	0.355016291
H	0.301770747	-2.098894200	-0.379544355
H	-2.353556771	-2.431212314	1.319931143
O	0.239154333	2.495454404	1.206879237
H	-0.517453462	2.445500914	0.228071671
H	0.626951714	3.364300029	1.351622641

28(ery) → **4et**+**7**

C	-2.427930032	-1.600258333	0.156271875
C	-1.749435470	-0.736248356	-0.786695742
C	-0.414112832	-0.081461436	-0.078773324
C	0.592838191	-1.183993762	0.280709523
O	-3.086702681	-1.153065959	1.129204719
O	-1.371327336	-1.398269822	-1.958689618
O	0.039565903	0.809845751	-0.988828002
O	1.707541328	-0.626989854	0.963069756
S	3.053790642	-0.204311130	-0.057903431
O	4.150022608	0.024480924	0.875575697
O	2.534628992	1.258747346	-0.523236350
H	-2.362851900	0.148780446	-0.989300732
H	-0.769142164	0.372787800	0.863229916
H	-2.316870959	-2.685803379	0.086809639

H	0.902333745	-1.714869806	-0.627978229
H	0.139577510	-1.912339102	0.965512867
H	-0.692365575	-0.819917453	-2.344554112
H	-0.963036973	1.966407951	-0.693065874
H	1.554395495	1.172946799	-0.784760351
O	-1.761689802	2.514067218	-0.353462867
H	-2.704187459	1.812253339	0.586362343
H	-2.068364968	3.072998463	-1.071996175
O	-3.287550342	1.265418195	1.239455197
H	-3.158386644	1.620787427	2.125548161
H	-3.174873697	-0.004471006	1.176328657

28(thr) → **4ec**+**7**

C	-2.083753299	1.682819067	0.459183686
C	-1.760300718	1.218006882	-0.857666080
C	-0.181068597	0.512548426	-0.663331567
C	0.859792115	1.572860832	-0.304319751
O	-2.602027651	0.980699938	1.369683873
O	-0.195700594	-0.484294291	0.199638420
O	2.174937133	1.042624322	-0.514897337
O	-3.230149638	-1.393977826	1.169164919
H	-1.750049565	2.673806583	0.775448804
H	-0.056403138	0.225033387	-1.727423330
H	0.736792457	1.870452817	0.743906146
H	0.810294034	2.456654062	-0.945692831
H	-0.869035344	-1.620851952	-0.327816074
H	-2.880232474	-0.044049929	1.145832568
H	-2.913743642	-1.784963783	1.991130567
O	-2.630112583	0.250842521	-1.358123841
H	-1.621709154	2.047683095	-1.556775252
O	-1.639485828	-2.228139585	-0.718795595
H	-2.749491239	-1.880209555	0.441737545
H	-1.283764340	-3.023281308	-1.122364792
S	2.602471380	-0.325842357	0.425093186
O	2.698565161	-1.439121760	-0.522307907
O	4.158862656	0.210652671	0.668332356
H	-2.224717706	-0.639165846	-1.376089519
H	4.618657318	0.206871051	-0.187592004

30(rib) → **31**(rib)

C	0.111809485	-1.610864926	0.126283107
O	-0.773815907	-0.761093982	-0.595048019
C	-1.932553888	0.182870758	0.704457301
C	-0.837636425	0.966052614	1.416078487
C	0.181911352	0.030683826	2.099921569
C	0.984662647	-0.854382671	1.129559506
O	-2.514958533	-0.697584845	1.391263289
O	-0.107754943	1.972127910	0.668457820

O	1.034799000	0.759454519	2.951105755
O	1.949941811	0.037731583	0.511418363
H	0.750470685	-2.154210727	-0.573008228
H	-0.469223661	-2.347849817	0.700148062
H	-2.445292723	0.672853100	-0.125027694
H	-1.346522608	1.516556323	2.215480769
H	-0.402302111	-0.635666081	2.738560881
H	1.540056619	-1.591222459	1.717450265
H	-3.496161173	-1.341018470	0.689185505
S	-0.342350752	2.517715718	-0.902627583
H	1.592269763	1.299564385	2.376941261
S	3.221610328	-0.563127929	-0.408312527
O	-2.525019870	-1.998360037	-1.790506444
H	-1.691608002	-1.481769167	-1.330997215
H	-2.241031356	-2.835370246	-2.169221529
O	-4.127675577	-1.870264109	-0.018821453
H	-4.456704063	-2.679880724	0.384315842
H	-3.402638217	-2.046098508	-0.947005250
O	-1.790856666	2.554778214	-1.158402117
O	0.275462821	1.251834230	-1.750834326
O	3.383884241	-1.991289523	-0.125063324
O	2.451348360	-0.528958108	-1.882384173
H	-0.148119930	0.383571253	-1.412914908
H	1.930507037	0.299041318	-1.955241032

32(rib) → **33**(rib)

O	-0.023519432	1.107624884	0.757255521
C	0.842892694	0.009142443	0.464770573
C	0.128779214	-0.841951707	-0.622310611
C	-1.324383916	-0.354080718	-0.617490327
C	-1.221424895	1.162511946	-0.454520706
C	2.214221721	0.503441354	-0.013312737
O	2.965587795	-0.558874998	-0.633098009
O	0.247462564	-2.243287847	-0.404565560
O	-1.958716777	-1.015106177	0.500905032
O	-0.802721442	1.774616754	-1.501475191
H	0.956300147	-0.590985221	1.371070874
H	0.558589589	-0.643351672	-1.602011074
H	-1.841081247	-0.597132749	-1.549086527
H	-2.007865997	1.613587414	0.166378146
H	2.792447171	0.927742155	0.812910175
H	2.080113373	1.249881699	-0.797811128
S	3.889606389	-1.581335194	0.349291175
H	-0.399943234	-2.473210439	0.278689337
S	-3.625610971	-1.280321116	0.386423020
H	-0.584877441	3.122796672	-1.328336069
O	-0.280497717	4.114036784	-1.019948830
H	-1.049654658	4.690042224	-0.980453490

H	0.436471686	2.275845981	0.971721829
O	0.690032733	3.392049283	1.062996778
H	0.271184000	3.849706758	0.120123613
H	1.637519315	3.544665011	1.139217668
O	4.330046561	-0.818497179	1.519450888
O	2.663066565	-2.541242431	0.919778365
O	-3.862221175	-2.270908210	1.432382766
O	-4.147191745	0.190641972	0.951743983
H	1.905232175	-2.585223271	0.290284070
H	-3.968680818	0.242243299	1.906976587
